# The role of plasmacytoid dendritic cells (pDCs) in immunity during viral infections and beyond

**DOI:** 10.1038/s41423-024-01167-5

**Published:** 2024-05-22

**Authors:** Clémence Ngo, Clémence Garrec, Elena Tomasello, Marc Dalod

**Affiliations:** Aix-Marseille University, CNRS, INSERM, CIML, Centre d’Immunologie de Marseille-Luminy, Turing Center for Living Systems, Marseille, France

**Keywords:** Interferon, Plasmacytoid dendritic cells, Viral infection, Tissue, Homeostasis, Infection, Innate immunity

## Abstract

Type I and III interferons (IFNs) are essential for antiviral immunity and act through two different but complimentary pathways. First, IFNs activate intracellular antimicrobial programs by triggering the upregulation of a broad repertoire of viral restriction factors. Second, IFNs activate innate and adaptive immunity. Dysregulation of IFN production can lead to severe immune system dysfunction. It is thus crucial to identify and characterize the cellular sources of IFNs, their effects, and their regulation to promote their beneficial effects and limit their detrimental effects, which can depend on the nature of the infected or diseased tissues, as we will discuss. Plasmacytoid dendritic cells (pDCs) can produce large amounts of all IFN subtypes during viral infection. pDCs are resistant to infection by many different viruses, thus inhibiting the immune evasion mechanisms of viruses that target IFN production or their downstream responses. Therefore, pDCs are considered essential for the control of viral infections and the establishment of protective immunity. A thorough bibliographical survey showed that, in most viral infections, despite being major IFN producers, pDCs are actually dispensable for host resistance, which is achieved by multiple IFN sources depending on the tissue. Moreover, primary innate and adaptive antiviral immune responses are only transiently affected in the absence of pDCs. More surprisingly, pDCs and their IFNs can be detrimental in some viral infections or autoimmune diseases. This makes the conservation of pDCs during vertebrate evolution an enigma and thus raises outstanding questions about their role not only in viral infections but also in other diseases and under physiological conditions.

## Introduction

Vertebrate antiviral immunity critically depends on type I and III interferons (IFNs) [1]. Indeed, mice with IFN receptor knockout and patients harboring homozygous loss-of-function polymorphisms in pathways promoting the induction of, or response to, IFNs suffer from severe viral infections [2–4]. IFN-Is include 13 members of the IFN-α family in humans (14 in mice), which are very structurally similar to one another: IFN-β, IFN-ε, IFN-κ, and either IFN-ω in humans or IFN-ξ in mice. The receptor for IFN-Is is the IFN-α receptor (IFNAR) [1]. IFNAR is expressed ubiquitously and is composed of two subunits, IFNAR1 and IFNAR2. IFN-IIIs are also called IFN-λ and include two members in mice and four in humans. All IFN-IIIs signal through a common heterodimeric receptor, the IFN-λ receptor (IFNLR), which consists of the IFNLR1 subunit (also called IL-28Rα) and the IL-10Rβ subunit. IFNLR is expressed selectively in epithelial cells as well as in certain hematopoietic cell types [1]. Consistently, IFN-IIIs are especially important for antiviral defense at epithelial barrier surfaces [5], particularly in the lung [6], gut [7], and female reproductive tract [8], as well as in the human liver [9]. IFN-IIIs exert antiviral effects that are very similar to those of IFN-Is. Vertebrate antiviral defenses are coordinated by IFNs via two complementary pathways [1]. First, IFNs exert direct antiviral effects by enforcing intrinsic antiviral immunity. Second, IFNs exert immunoregulatory effects that can promote protective innate and adaptive antiviral immunity. Intrinsic antiviral immunity is a group of cell-autonomous defense mechanisms mediated by molecules that are constitutively expressed and, in most cases, act as restriction factors able to inhibit a particular stage of the viral life cycle in the target cell, from entry through genome replication to budding [1]. Many of the genes stimulated by IFNs (IFN-stimulated genes or ISGs) encode viral restriction factors. Hence, IFNs enforce intrinsic antiviral immunity by further increasing the level of expression of their effector molecules in the vast majority of the cells of the body [1]. In addition, IFN-Is can modulate the functions of a broad spectrum of innate and adaptive immune cells [1]. In particular, during viral infections, IFN-Is constitute one of the most important input signals acting on dendritic cells (DCs) to promote their delivery of appropriate output signals to T cells, B cells and natural killer (NK) cells for protective immunity [1]. Moreover, IFN-Is can directly promote the activation of the effector functions of antiviral NK and CD8+ T cells and the production of antibodies by B cells. Hence, IFNs play key roles in the orchestration of protective responses of both innate and adaptive immune cells during viral infections [1].

IFNs can also exert a variety of deleterious effects on the host. For example, IFN-Is can inappropriately exacerbate immune responses, which in turn can increase host susceptibility to bacterial infections or even to some chronic viral infections [1]. IFN-III production in infected lungs can delay or compromise epithelial cell repair, leading to enhanced susceptibility to secondary bacterial infections [10, 11]. Abnormally high and/or chronic production of IFN-Is strongly contributes to the development of various autoimmune or inflammatory diseases [1]. Hence, IFN production and responses must be tightly regulated under physiological conditions and during viral infections to avoid unbridled inflammation leading to tissue damage and eventually autoimmunity. Therefore, a better understanding of the cellular and molecular mechanisms controlling IFN production during viral infections is important for designing novel strategies to manipulate these responses in a manner that promotes their protective functions while preventing their deleterious effects, depending on the pathophysiological context, including the diseased tissue.

**Fig. 1:**
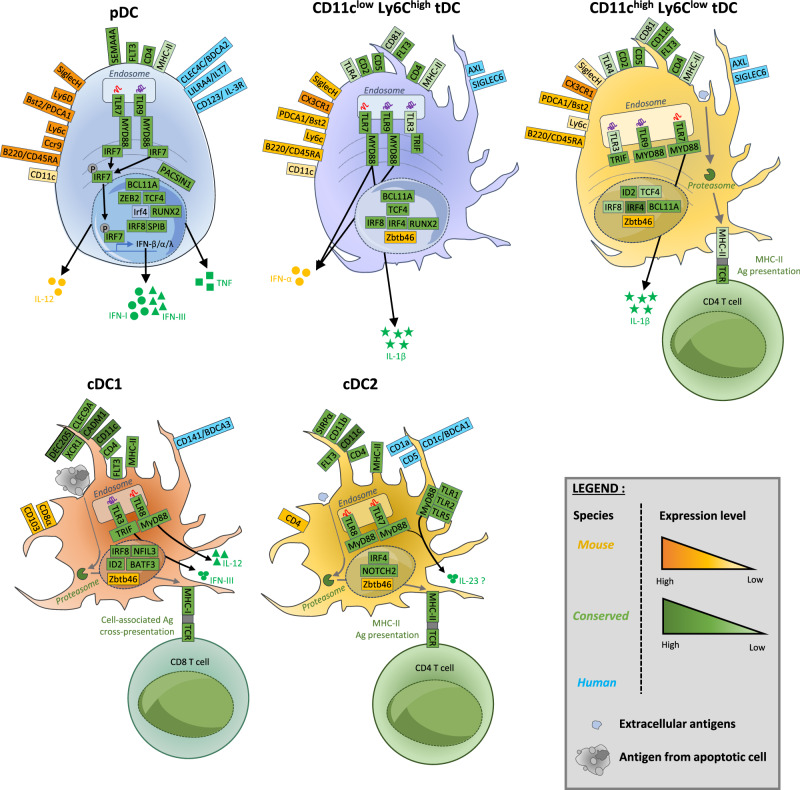
Phenotypic and functional description of dendritic cell types. Dendritic cells (DCs) encompass different cell types, including plasmacytoid dendritic cells (pDCs), CD11c^low^ (CD11c^low^ Ly6C^high^) and CD11c^high^ (CD11c^high^ Ly6C^low^) transitional dendritic cells (tDCs) and conventional dendritic cells (cDCs), which are divided into type 1 cDCs (cDC1s) and type 2 cDCs (cDC2s). pDCs are characterized by their capacity to produce large amounts of IFN-I/IIIs upon exposure to a large spectrum of TLR7/9 ligands of viral or synthetic origin (e.g., CpG A and B), while CD11c^low^ Ly6C^high^ tDCs are activated mainly by CpG-B. IFN-IIIs can also be produced by cDC1s via a TLR3-TRIF-dependentmechanism. At steady state, cDC2s and CD11c^high^ Ly6C^low^ tDCs can present antigens (pale blue) associated with MHC class II (MHC-II) for CD4 T-cell activation, while cDC1s excel in the ability to cross-present cell-associated antigens for CD8 T-cell activation (gray). Upon activation, pDCs also acquire the transcriptional, phenotypic, and functional features of antigen-presenting cells. However, their ability to contribute to the antigen-specific activation of T cells in vivo is controversial. The expression of selected cell surface, cytoplasmic and endosomal molecules, as well as some of the key nuclear transcription factors controlling their development or functions, is shown for each DC type. Molecules exclusively expressed in mice or in humans are depicted in yellow or blue, respectively, while molecules conserved between the two species are in green. The color intensity is proportional to the level of expression

A major cellular source of IFN-Is and IFN-IIIs is plasmacytoid dendritic cells (pDCs) (Fig. 1), particularly during systemic viral infections and certain autoimmune diseases characterized by high expression of ISGs called interferonopathies [1]. However, following infection by a virus, virtually any type of nucleated cell can produce IFNs via cytoplasmic sensing of viral-derived nucleotide sequences by dedicated helicases, triggering the activation of the adaptor stimulator of IFN genes (STING) and downstream phosphorylation of IRF3 [1]. In addition, type 1 conventional DCs (cDC1s) (Fig. 1), a type of dendritic cell that specializes in the highly efficient activation of cytotoxic CD8+ T cells, including through cross-presentation of cell-associated antigens, can produce high levels of IFN-IIIs in response to certain viral-type stimuli [1]. Thus, during viral infections, different pathways can promote the production of IFNs in the host, raising the question of their redundancy or complementarity. In this regard, we will review the biology of pDCs, their IFN production in vivo during viral infections, their regulation in different tissues, and their contribution to the induction of IFN responses and, more generally, to host resistance. This report will help provide a deeper understanding of pDC functions, including their role in tissue homeostasis. We will conclude with the remaining outstanding questions regarding the role of pDCs during viral infections and beyond in different tissues, and we will discuss possible strategies to answer these questions in future studies.

## Generalities of pDCs based on their identification and study in vitro

### pDCs are professional producers of type I interferons that respond to many viruses

It is well known that all nucleated cells can produce IFN-Is in response to viral infection. However, in the late 1970s and early 1980s, it was discovered that, in human peripheral blood, rare cells expressing major histocompatibility complex class II molecules exhibited the unique ability to rapidly produce much greater amounts of IFN-Is in vitro in response to a much broader variety of viruses than other cell types [12, 13]. These cells were hence termed “natural IFN-producing cells” (NIPCs or IPCs). It was only in 1999 that their elusive nature was solved in parallel by two teams, who succeeded in isolating them and further characterizing their phenotype [14, 15]. The mouse equivalent of human pDCs was identified shortly after, in parallel by several teams, based on their unique ability to produce massive amounts of IFN-Is in vitro in response to viruses [16–18]. Hence, the most important defining feature of pDCs is that they are professional producers of IFN-Is in response to many viruses (Fig. 1).

### pDCs exhibit a plasmacytoid morphology at the steady state but can acquire a dendritic morphology and can activate naïve T cells upon adequate stimulation

Due to their plasmacytoid morphology at steady state (Fig. 1) and their ability to acquire a dendritic morphology and activate naïve T cells in a cognate manner in vitro, both in humans [19, 20] and in mice [21, 22], NIPCs were renamed plasmacytoid predendritic cells [23], which was later simplified into the current nomenclature of pDCs. No single cell surface marker is sufficient to identify pDCs, either in humans or mice. In mice, pDCs can be characterized as CD11b^−^, CD11c^int^, Ly6D^+^, Bst2^high^, SiglecH^+^ and CCR9^+^ [24]. In humans, pDCs can be characterized as CD11c^-^, CD33^-^, CD123 (IL-3R)^+^, CLEC4C (BDCA2)^+^ and LILRA4 (ILT7)^+^ [25, 26]. pDCs express PACSIN1, MHC-II, TLR7, TLR9, IRF7, SPIB, TCF4, RUNX2 and ZEB2 in both mice and humans [27, 28] (Fig. 1).

### pDCs strongly depend on IRF7 for their robust production of all IFN-I subtypes

The two master transcription factors driving IFN-I expression are interferon regulatory factor (IRF) 3 and 7. In most cells, IRF3 is constitutively expressed, whereas IRF7 is induced in response to IFN-Is. IFN-β can be induced by IRF3, whereas the expression of most IFN-α subtypes requires IRF7. As a consequence, infected cells mostly produce IFN-β, and only a very restricted set of IFN-α subtypes and at relatively low levels. In contrast, because they express IRF7 constitutively [29] and with heightened protein stability [30], pDCs can produce all subtypes of IFN-Is quickly and in high quantities, a function to which they dedicate up to 60% of their new transcriptional activity at their activation peak [31]. As a corollary, high IFN-I production by pDCs strictly depends on IRF7 [32–34] (Fig. 1).

### pDCs sense viral nucleic acids via endosomal Toll-like receptors 7 and 9

Soon after the discovery of Toll-like receptors (TLRs) as innate immune sensors able to recognize pathogen-associated molecular patterns and danger-associated molecular patterns[35–37], TLR9 was shown to recognize unmethylated CpG DNA sequences [38] and activate pDC IFN-I production [39]. Moreover, transcriptional profiling of their expression across human cell types revealed the selective expression of TLR7 and TLR9 in pDCs and B cells [40]. Hence, TLR7 and TLR9 were identified as likely candidates for innate sensing of viral-derived nucleic acids by pDCs. This was indeed proven to be the case both in mice [41–43] and humans [44]. Hence, the professional IFN-I production capacity of pDCs depends on their ability to sense viral-derived nucleic acids and the induction of the downstream signaling cascade TLR7/9→MYD88→IRF7 (Fig. 1).

### pDCs are differentially and more robustly activated by infected cells than by free viral particles

**Fig. 2:**
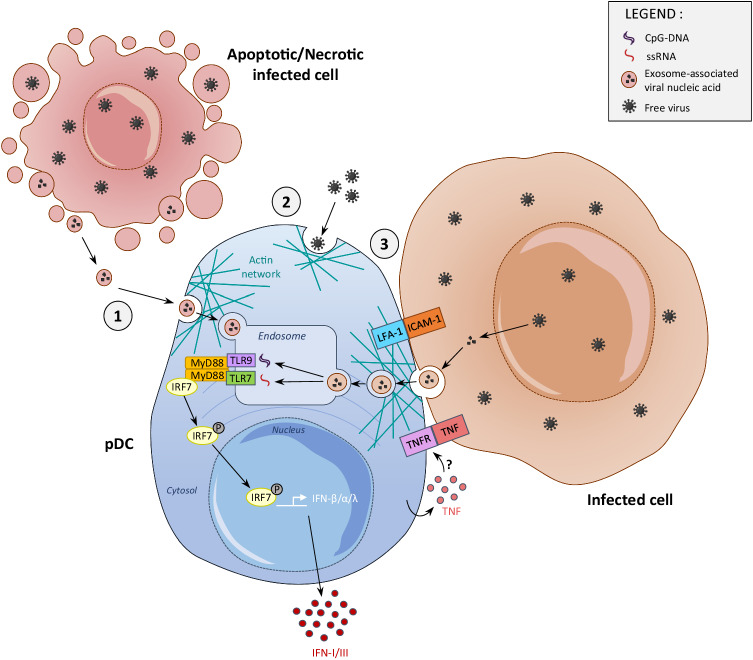
Molecular mechanisms of viral sensing by pDCs. pDCs sense viral nucleic acids through endosomal TLR7 and TLR9, which recognize single-stranded RNA rich in uridine and unmethylated CpG DNA, respectively. When endosomal TLR7/9 interact with their respective ligands, the MYD88-IRF7 signaling pathway is activated. This leads to the recruitment and phosphorylation of the transcription factor interferon regulatory Factor 7 (IRF7), which is translocated to the nucleus, where it induces transcription of the genes encoding IFN-α/β (IFN-Is) and IFN-λ (IFN-IIIs). Different viral recognition mechanisms have been proposed to promote pDC activation and trigger IFN production. Virus-derived nucleic acid contained in exosomes or apoptotic/necrotic bodies released from infected cells can be captured by pDCs and engulfed into endosomes ①. Free viruses can also be captured by pDCs via unknown receptors and activate them ②. Finally, pDCs can establish contact-dependent interactions with infected cells, generating an immune synapse involving adhesion molecules, such as LFA-1, which is expressed by pDCs, and ICAM-1, which is expressed by infected cells ③. The TNFR expressed by pDCs may also stabilize this synapse upon interaction with its ligand TNF, which can be expressed in a membrane-bound form at the surface of infected cells. As another source of TNF, pDCs may also amplify their own IFN production in an autocrine or paracrine response, but other TNF sources may also be involved. The stabilization of the immune synapse requires the polarization of the actin network in pDCs, which enables pDCs to capture viral material, the nature of which has not yet been elucidated and could vary depending on the nature of the virus and the infected cells

The ability of pDCs to produce IFNs in response to viral stimulation in vitro was initially studied upon exposure of human blood or mouse splenic pDCs to free viral particles [14–18]. However, it was later discovered that cells infected with DNA or RNA viruses induce more intense, prolonged and/or diverse production of IFN-I/IIIs by pDCs, both for mouse pDCs infected with vesicular stomatitis virus (VSV) [45] and for human pDCs infected with human immunodeficiency virus type 1 (HIV-1) [46], human cytomegalovirus (HCMV) [47], influenza A virus (IAV) [47] or severe acute respiratory syndrome coronavirus 2 (SARS-CoV-2) [48, 49]. This is also true for pDCs of other mammalian species, such as pigs, when exposed to cells infected by porcine reproductive and respiratory syndrome virus (PRRSV) [50] or classical swine fever virus (CSFV) [51]. Mechanistically, pDCs establish prolonged cell contact-dependent interactions with infected cells via the generation of an interferogenic synapse, as observed with human pDCs exposed to cells infected with herpes simplex virus 1 (HSV-1) [52], hepatitis C virus (HCV), human T-lymphotropic virus (HTLV), dengue virus (DENV) and Zika virus (ZIKV) [53–56]. This process involves first the engagement of complementary adhesion molecules expressed at the surface of the interacting partners, namely, the integrin molecule LFA-1 on pDCs and ICAM-1 on infected cells [47, 56], leading to the polarization of the pDC actin network close to the contact site and promoting the formation of a stable immune synapse [56] (Fig. 2). This process allows pDCs to capture viral material from infected cells for intracellular trafficking to dedicated endosomes where viral-derived nucleic acids trigger the TLR7-9→MYD88→IRF7 signaling pathway, which induces IFN production [56]. Inhibition of the endosomal sorting complex required for transport (ESCRT) machinery in virally infected cells impairs pDC activation [53]. The nature of the viral cargo delivered from infected cells to pDCs can vary depending on both the virus and the type of infected cell; it can encompass exosomes containing viral RNA [50, 53], immature viral particles [54] or viral biofilms [55]. The identity of the pDC receptors involved in the capture and transport of viral material has not yet been elucidated.

### pDCs are generally resistant to viral infection

Human blood pDCs have been found to be resistant to infection with different DNA or RNA viruses, including IAV, Middle East respiratory syndrome coronavirus (MERS-CoV), SARS-CoV-2, VSV, HSV-1, and HIV-1 in vitro [14, 48, 57–59]. The resistance of pDCs to viral infections can vary depending on both the host and the virus. Indeed, Bluetongue virus (BTV) replicates in vitro in sheep pDCs that produce IFN-Is via Myd88-independent pathways [60, 61]. Resistance of pDCs to productive viral infection has been proposed to result from their high basal expression of ISGs, including IRF7, and high IFN production [62]. However, IFN-I blockade does not increase human pDC sensitivity to infection by HIV-1 in vitro [58]. IRF-1-dependent mechanisms could be involved in pDC resistance to viral infection, as shown in bone marrow (BM)-derived pDCs infected in vitro with VSV [45].

In summary, in vitro studies have clearly demonstrated the unique ability of pDCs to sense most viruses and rapidly produce massive amounts of all subtypes of IFN-I/IIIs. pDCs are resistant to infections by many viruses but engulf viral particles or material derived from infected cells and route these cargos to dedicated endosomes to trigger the TLR7/9-to-MYD88-to-IRF7 signaling pathway, which activates the promoters of all IFN-I/III genes (Fig. 2). Hence, pDCs escape the effects of viral immune evasion genes that evolved to prevent IFN-I/III production in infected cells. This is exemplified by the fact that cDCs infected by a wild-type strain of IAV hardly produce any IFN-I, whereas upon infection with a mutant strain that is unable to interfere with viral mRNA sensing by host cells via its NS1 protein, they become high producers [63]. Due to these combined properties, pDCs are considered to be critical for host defense against viral infections by inducing a broad IFN-dependent infection-resistant state upon reinforcement of antiviral intrinsic immunity in virtually all host cells [62, 64]. We will further discuss this hypothesis, taking into consideration the limitations of the experimental tools used to selectively deplete pDCs, perturb their IFN-I production (Table 1) [16, 65–75] or visualize them in vivo (Table 2) [24, 70, 76–79]. Our extensive analysis of the literature aimed at deciphering the physiological functions of pDCs during in vivo viral infections is summarized in Table 3 [16, 21, 24, 41, 65, 70, 71, 79–104] and Table 4 [92, 96, 105–123].

**Table 1:** Tools currently used to deplete pDCs or to inhibit their IFN production

Mutant mice	Genetic background	Principle/Advantage	Penetrance^1^	tDCs impacted	Other cell types impacted
*CX3CR1*-hDTR [65]	C57BL/6	Depletion of precursors common to pDCs, tDCs and cDCs	>90%	YES	cDC2
CD11c-Cre; *Tcf4*^-/fl^ (CKO) [66]	C57BL/6	Loss of Tcf4 exclusively in CD11c^+^ cells, defect in pDC and tDC development	>90%	YES	ND^2^
*Tcf4*^-/-^ [66]	129SvEvTac	defect in pDC development	>90%	YES	tDCs, pDC-like cells, monocytes, macrophages, and subsets of B cells
*Ikaros*^L/L^ [67]	C57BL/6	Hypomorphic *Ikaros* mutation, defect in pDC development	95%	ND	B cells, T cells, neutrophils
*Siglech*-hDTR [68]	C57BL/6	Bacterial artificial chromosome-based transgenic mice expressing hDTR under the control of *Siglech* regulatory regions	90-95%	ND	Marginal Zone Macrophages (MZM), pDC precursors and other specialized macrophages (e.g. microglia)
*Siglech*-hDTR [69]	C57BL/6	KI mice expressing hDTR under the control of the *Siglech* promoter	95%	ND	ND
CLEC4C-hDTR/BDCA2-hDTR [70]	C57BL/6	Transgenic mice expressing hDTR under the control of human *CLEC4C/BDCA2* regulatory regions	90-95%	NO	NO
**Antigen** + **(antibody clone)**	**Targeted host**	**Principle/Advantage**	**Penetrance**	**Are tDCs impacted?**	**Other cell types impacted?**
Ly6C/Ly6G (RB6-8C5) [16, 71]	Mouse	Depletion of pDCs as they express high levels of Ly6C	95%	ND	Neutrophils, monocytes, eosinophils, macrophages and activated T cells
Bst2 (120G8 or 927 Ab clones) [65, 72]		Depletion of pDCs as they express high levels of Bst2	94%	YES	tDCs, activated monocytes and B cells
SiglecH (440c Ab clone) [73]		Inhibition of IFN production by pDCs	90%	ND	Macrophages and DC progenitors
BDCA2 (Litifilimab) [74]	Human and Macaques	Depletion of pDCs as they express BDCA2	Data not shown	ND	ND
ILT7 (VIB7734) [75]		Depletion of pDCs as they express ILT7	50% in human, 80% in macaque	NO	ND

^1^Percent of the pDC population that is depleted, or in the case of anti-SiglecH administration, percent of inhibition of pDC IFN production; ^2^ND not determined

**Table 2:** Mutant mice currently available and used to visualize pDCs

Model	Principle/Advantage	Penetrance^1^	tDCs tracing	Other cell types tracing
pDC-Tom (*Siglech*^iCre^; *Pacsin1*^LSL-tdT^) [24]	Model of intersectional genetics based on exclusive coexpression of *Siglech* and *Pacsin1* in pDCs. *Siglech*^iCre^ remove a loxP-STOP-loxP sequence upstream of a gene encoding tdTomato (tdT) reporter located 3’ of the *Pacsin1* gene. Only pDCs express tdT.	80%	NO	NO
SCRIPT (*Siglech*^iCre^ ; *Ifnb1*^EYFP^; *Pacsin1*^LSL-tdT^) [24]	Crossing of pDC-Tom mice with *Ifnb1*^*EYFP*^ mice allow to discriminating IFN-producing pDCs (tdT+ YFP + ) from nonproducing ones (tdT+ YFP-)	80%	NO	NO
ZeST (*Zbtb46*^GFP^; *Siglech*^iCre^; *Pacsin1*^LSL-tdT^) [24]	Crossing of pDC-Tom mice with *Zbtb46*^*GFP*^ mice allows discriminating pDCs (tdT^+^ GFP^-^) from tDCs and cDCs (tdT^-^ YFP^+^)	80%	YES	YES (cDC and DC precusors)
DPE-GFP [76]	The gene encoding GFP is under the control of the distal and proximal enhancers and promoter of mouse *Cd4* gene	95%	YES	T cells
DPE-GFP x *Rag1*-KO [77]	Crossing DPE-GFP with *Rag1*-KO mice results in GFP expression exclusively in CD4^+^ myeloid cells	95%	YES	YES
*Siglech*-GFP [70]	The gene encoding GFP is under the control of the *Siglech* gene	90%	N.D.	YES (SiglecH^+^ macrophages)
pDCre-tg [78]	The gene encoding the Cre recombinase is under the control of a BAC sequence containing the *Siglech* gene	30%	YES	YES (DCs, DC precursors, T, B, NK cells)
*Siglech*^iCre^ [79]	The gene encoding the Cre recombinase is under the control of the endogenous *Siglech* gene	95%	YES	YES (DCs, DC precursors, T, B, NK cells)

^1^Percent of the pDC population that is traced

**Table 3:** pDC activation and functions during systemic viral infections

Virus; host	pDC infection or exhaustion	pDC contribution to antiviral immune functions			
		IFN-I production		Host resistance	Intrinsic, innate or adaptive immunity
		Contribution^1^	Site; method^2^		
MCMV; mouse	Most pDCs not infected, including IFN-producing pDCs [21, 79]	Major [16, 24, 41, 80–83]	Serum; ELISA [16, 41, 71, 80, 82] Spleen; qRT‒PCR [81], FC and IF using anti-IFN-α/β Ab or *Ifnb1*^EYFP^ mice [24, 79, 81, 83]	Dispensable pDC depletion did not increase mortality in BDCA2-hDTR or anti-Bst2-treated mice [70, 82]	Dispensable for intrinsic immunity, splenic ISG induction maintained in anti-Bst2-treated or *Myd88*-KO mice [82] Contributes to innate immunity, pDC depletion in BDCA2-hDTR or anti-Bst2-treated mice decreased cDC1 and NK cell activation [16, 41, 70, 71, 82] Putatively contributing to adaptive immunity, in vivo activated pDCs become able to activate naïve T cells upon antigenic pulse ex vivo [21, 83].
VSV; mouse	ND	Minor [70]	Serum; ELISA	Minor pDC depletion in BDCA2-hDTR mice increased viral titers only transiently [70]	Contributes to adaptive immunity, pDC depletion in BDCA2-hDTR mice reduced CD8 T-cell numbers and survival [70].
LCMV, acute; mouse	ND	Minor [71, 84–86]	Spleen; qRT‒PCR and IFN-I titration on B220^+/-^ CD11c^+^ cells [84], FC using *Ifna6*^GFP^ mice [85] Serum; ELISA [71, 86]	Minor pDC depletion in BDCA2-hDTR mice increased viral titers only transiently [86]. Tlr7^-/-^Tlr9^-/-^ mice did not exhibit increased mortality [85]	Controversial role in promoting adaptive immunity. Decreased responses in *Tlr7*^-/-^*Tlr9*^-/-^ mice but whether this was pDC-dependent was not established [85]. Depletion of BST2^+^ cells compromised antigen-specific activation of CD8 T cells during infection only in mice deficient for cytosolic sensing of viral replication, together with other data this suggested a prominent role of cDCs over pDCs for IFN production and adaptive immunity induction [87].
LCMV, chronic; mouse	IFN-producing pDCs not infected; other pDCs can be infected [88] pDC exhaustion [88, 89]	Major only early after infection [71, 84–86, 88]	Spleen; FC using *Ifnb1*^EYFP^ mice [88] Serum; ELISA [86]	Minor early after infection, pDC depletion in BDCA2-hDTR mice increased viral titers only very slightly and transiently [86] Contributes to the chronic infection phase, CKO mice^3^ exhibited increased viral titers [90]	Controversial role in adaptive immunity. Impaired CD4 T-cell activation in CKO mice [90]. pDC depletion in BDCA2-hDTR mice did not affect antiviral CD8 T-cell responses and viral control at least until 30 days after infection [86]. Tlr7-deficiency impaired CD8 T-cell antiviral functionality as early as day 8 and prevented viral clearance between days 70 and 120, but whether this was pDC-dependent was not established [91].
HSV-1; mouse	ND	Major [92]	Serum; ELISA	ND	Contributes to innate and adaptive immunity, NK and CD8 T-cell activation was reduced upon pDC depletion in BDCA2-hDTR mice [92].
HSV-2; mouse	ND	Major [92]	Serum; ELISA	Major, increased mortality upon pDC depletion in BDCA2-hDTR mice [92]	Dispensable.
MHV; mouse	ND	Major [65, 90, 93, 94]	Serum; ELISA Spleen; qRT‒PCR [93]	Minor, pDC depletion in BDCA2-hDTR, CX3CR1-hDTR or anti-Bst2-treated mice increased viral titers and serum ALT levels, but much less than in *Ifnar1*-KO mice [65, 90, 93] Decreased survival of DT-treated BDCA2-DTR mice, due to enhanced IL-1β-dependent deleterious role of tDCs rather than loss of pDC IFN production [65]	Contributes to immunity, increased numbers of monocytes and neutrophils upon pDC depletion in BDCA2-hDTR mice [65].
NDV; mouse	WT pDCs not infected; *Ifnar1*-KO pDCs infected [95]	Major [95, 96]	Serum; ELISA Spleen; FC or IF using *Ifna6*^GFP^ mice [95, 96]	Dispensable	ND
FMDV; cattle	ND	Major [97]	Serum; ELISA	ND	ND
SIV; macaque HIV; human	pDCs not infected Death of activated pDCs, replaced by exhausted precursors in macaques [98] Exhaustion of human pDCs [99]	Major in acute phase Controversial in chronic phase [98, 100–104] Major upon viral rebound after treatment interruption [99]	Serum; ELISA High in spleen and LNs, moderate in intestine, undetectable in blood; intracellular FC	ND	ND

^1^Major, pDCs were a major source of IFN-Is. Minor, pDCs were a minor source of IFN-Is; ^2^FC flow cytometry, IF immunofluorescence, qRT‒PCR quantitative reverse transcriptase‒polymerase chain reaction. ^3^CKO mice are CD11c-Cre;*Tcf4*^-/fl^animals (see Table 1). ND not determined, LN lymph node, BAL bronchoalveolar lavage

**Table 4:** pDC activation and functions during local viral infections

Virus; host	Infection route	pDC contribution to antiviral immune functions			
		IFN-I production		Host resistance	Intrinsic, innate or adaptive immunity
		Contribution^1^	Site; method^2^		
VSV; mouse	footpad	Major, with infected SCM [105]	LN homogenate; ELISA	Contributes to immunity, increased VSV propagation from popliteal to inguinal LN upon pDC depletion in DT-treated BDCA2-hDTR mice [106]	ND
MVA; mouse	footpad	Minor [106]	LN homogenate; ELISA	ND	Contributes to adaptive immunity, reduced CD8 T-cell numbers and impaired cDC1 activation upon pDC depletion in BDCA2-hDTR mice [106]
ECTV; mouse		Minor [107]	LNs; intracellular FC	Putative protective role, slighty decreased survival upon Bst2^+^ cell depletion	ND
HSV-1; mouse	Intracorneal	Major [108]	Cornea; qRT‒PCR for *Ifna*	Major protective role, increased viral load, morbidity and mortality upon pDC depletion in BDCA2-hDTR mice [108]	Reinforcement of intrinsic immunity, corneal nerve infection and corneal homogenate viral titers are increased already on day 1 postinfection upon local pDC depletion in BDCA2-hDTR mice Contributes to adaptive immunity, pDCs preserve CD4 Tregs in the draining LN [108]
IAV; mouse	intranasal	Controversial Major [109–113] Minor [114]	Spleen homogenate [110], lung homogenate [109, 112], lung [111, 113, 115, 116]; BAL [111, 114]; ELISA, qRT‒PCR [111, 113, 115, 116]	Controversial Putatively protective role, Bst2^+^ cell depletion increased viral titers in Mx1^+^ C57BL/6 mice [117] Putatively deleterious role, Bst2^+^ cell depletion slightly decreased viral titers in Mx1^-/-^ BALB/c mice [112, 115] and reduced their morbidity and mortality [115]; Bst2^+^ cell depletion reduced the morbidity and mortality of 129S mice [111, 116] Redundant role, lack of pDCs in *Ikaros*^*L/L*^ or anti-Bst2-treated mice did not change viral titers and survival [110, 114]	Putatively contributes to innate immunity and inflammation, with contrasting results in anti-Bst2-treated mice: higher numbers of monocytes and production of TNF and IL-6 [112] versus reduced numbers of monocytes and production of inflammatory cytokines [111, 116] Putatively contributes to modulate adaptive immunity: reduction of anti-IAV antibodies in anti-Bst2-treated C57BL/6 [114], delay in CD8 T-cell recruitment in *Ikaros*^*L/L*^ mice [110], possible induction of IAV-specific CD8 T-cell apoptosis in the draining LN by FASL^+^ pDCs [115]
SARS-CoV1; mouse	intranasal	Major [118]	Ex vivo isolated lung Siglech^+^ cells; qRT‒PCR for *Ifna* and *Ifnb1* [118]	Putative deleterious role, Bst2^+^ cell depletion diminishes lung lesions and inflammation while increasing survival	ND
MERS-CoV; mouse	intranasal	Major [119]	Lung; qRT‒PCR (indirect evidence: decrease in *Ifna* and *Ifnb1* expression in *Tlr7*-KO mice) [119]	ND, but it is predicted to depend on the kinetics of pDC IFN-I production, since administration of exogenous IFN-β is beneficial early (between 6 h and 24 h postinfection) but deleterious later (at 2 days or 4 days postinfection) [119]	ND
NDV; mouse	intranasal	Minor [96, 113]	lung; FC using *Ifna6*^GFP^ mice [96, 113]	ND	ND
RSV; mouse	intranasal	Minor [109]	Lung; ELISA, in situ hybridization with *Ifna4* probe	ND	ND
PVM; mouse	intranasal	Major [120]	BAL, ELISA	Beneficial role. pDC depletion in BDCA2-hDTR neonates increased viral load and bronchiolitis, as well as predisposition to asthma upon reinfection in adulthood.	Contributes to immunity. pDC depletion in BDCA2-hDTR mice increased pro-inflammatory neutrophils, eosinophils and cytokines, and reduced NRP^+^ Treg [120]
MCV; human	cutaneous	Contributing [121]	Skin biopsies; qRT‒PCR for *Ifna*	ND	ND
HSV-2; mouse	vaginal	Minor [92]	Vaginal homogenate; ELISA	No difference in viral titers and mouse survival upon pDC depletion in BDCA2-DTR mice [92]	ND
RV; mouse, piglet, human	Gut	Putatively major [122, 123]	In vitro; intracellular FC of RV-stimulated human PBMCs, IFN neutralization in RV-stimulated mouse pDC/B-cell cocultures [122] Intestinal cells from infected piglets; intracellular FC [123]	Protective role, increased viral shedding upon pDC depletion in anti-Bst2-treated or BDCA2-DTR mice, or upon in vivo inhibition of pDC IFN-I production upon anti-Siglech Ab administration [122]	Promotion of antiviral IgG and IgA antibodies in the gut, since these responses are decreased upon pDC depletion or inhibition, explaining increased viral shedding in these experimental conditions [122]

^1^Major, pDCs were a major source of IFN-Is. Minor, pDCs were a minor source of IFN-Is; ^2^FC flow cytometry, qRT‒PCR quantitative reverse transcriptase‒polymerase chain reaction. SCM subcapsular sinus macrophages, ND not determined, LN lymph node, BAL bronchoalveolar lavage

## Are pDCs a major source of IFNs during viral infections in vivo, and in which tissues?

**Fig. 3:**
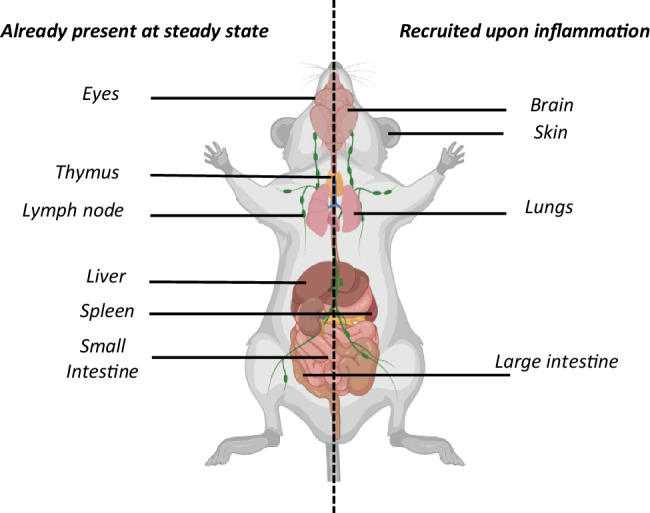
Resident and recruited pDC populations in the whole mouse body. At steady state, pDCs are widely distributed in the body, located in the indicated lymphoid organs (thymus, lymph node, spleen) and nonlymphoid tissues (eyes, liver, spleen and small intestine), as depicted here in mice. However, upon inflammation, pDCs can be recruited to other tissues (e.g., the brain, skin, lungs and large intestine), where their functions differ depending on the pathophysiological context. This knowledge should be extended in the future by performing whole-body cartography of pDC distribution using novel mouse models or tools allowing specific pDC detection in situ

At steady state, pDCs are located in several lymphoid organs (e.g., the spleen, lymph nodes, and thymus) and nonlymphoid tissues (e.g., the eye, liver, and small intestine). Upon viral infection or stimulation by other inflammatory factors, they can be recruited to other organs, such as the brain, skin, lungs and large intestine (Fig. 3). Next, we summarize the main findings regarding the role of pDCs in systemic and local viral infections affecting these organs.

### pDCs are the major source of IFN-Is during many but not all systemic viral infections

The first analysis of the contribution of pDCs to IFN production during viral infections in vivo was carried out in mice that were intraperitoneally injected with two natural rodent pathogens, Mouse Cytomegalovirus (MCMV) or lymphocytic choriomeningitis virus (LCMV) [16, 71], leading to acute systemic infections (Table 3). pDCs were shown to be a major source of IFNs during systemic MCMV but not LCMV infection by performing a combination of complementary experiments, including the measurement of ex vivo cytokine production by cell types purified from the spleens of infected mice and the analysis of the impact of in vivo administration of an anti-GR1 antibody on the serum IFN levels of cells expressing Ly6C or Ly6G, which encompasses pDCs as well as classical monocytes and neutrophils (Table 1).

The major contribution of spleen pDCs to IFN production during systemic MCMV infection was confirmed by different teams and using progressively refined methods allowing to identify and target pDCs with increased specificity (Table 3), including (i) treatment with an anti-PDCA1/Bst2 antibody [41], which depletes pDCs as well as fractions of other cell types including plasma cells, transitional DCs (tDCs) (Fig. 1), activated B cells, DCs and monocytes [72] (Table 1), (ii) analyses of IFN levels in serum and of ex vivo IFN production by sorted splenic cell populations from mice knocked-out for *Myd88*, *Tlr9* or *Tlr7* [41, 42, 80, 124], which mediate additional functions besides being required for pDC IFN-I production in response to viruses (Table 1), (iii) ex vivo flow cytometry analysis of intracellular IFN expression in cell suspensions from different organs of infected mice [81], (iv) confocal microscopy for IFN and pDC staining on spleen sections [79, 81], (v) flow cytometry and confocal microscopy analyses using *Ifnb1*^EYFP^ reporter mice [83] or more recently, double reporter mice enabling to monitor *Ifnb1* expression and simultaneously to specifically identify and visualize pDCs [24] (Table 2), and (vi) diphtheria toxin treatment in BDCA2-hDTR mice to efficiently and specifically deplete pDCs (Table 1) [70].

pDCs are also the main source of IFN-Is during mouse systemic infection with herpes simplex virus type 1 and 2 (HSV-1 and HSV-2) (Table 3), as assessed using BDCA2-hDTR mice [92] (Table 1), and with the coronavirus mouse hepatitis virus (MHV), as assessed upon depletion with an anti-PDCA1 antibody [90, 93] or in mutant CD11c-Cre;Tcf4^-/fl^ (CKO) mice constitutively devoid of pDCs [90] and tDCs [65, 94] (Tables 1 and 3). pDCs are also the main source of IFN-Is in cattle infected with Foot-and-Mouth Disease Virus (FMDV), as supported by the strong decrease in serum IFN levels upon in vivo depletion of CD4 + T cells (Table 3) [97]. pDCs were also shown to be a major source of IFNs during the acute phase of systemic infection of macaques with simian immunodeficiency virus (SIV) (Table 3) [98]. The contribution of pDCs to IFN production during the chronic phase of SIV infection is controversial, with a lack of detection by intracellular staining of cells isolated from lymph node biopsies in one study [98] versus specific detection of IFN transcripts in pooled pDCs isolated from the lymph nodes at necropsy in another study [125]. This controversy illustrates a possible sensitivity threshold issue, whereby it might be technically difficult to detect IFN-producing pDCs when their frequency is very low and a limited number of cells are analyzed.

### During systemic viral infections, IFN-I production by pDCs occurs primarily in the spleen and eventually in the lymph nodes but not in the blood

Although MCMV spreads to several lymphoid and nonlymphoid organs containing pDCs, most of the circulating IFN-Is are produced in the spleen (Table 3). A much lower fraction of IFN-producing pDCs was observed in the bone marrow, and no IFN-producing pDCs were observed in the blood, lymph nodes (LNs), lungs or liver [81]. During acute SIV infection in macaques, IFN-I production by pDCs was also prominently observed in the spleen, as well as in the LNs and, to a lesser extent, in the intestine but not in the blood (Table 3) [98]. In a study of chronic SIV infection in macaques, IFN transcripts were detected in LN pDCs, but this was not the case for blood pDCs [125]. In humans chronically infected by HIV-1, IFN-producing pDCs were not detected in the blood but were detected in the LNs in at least one study [126]. It is likely that the lack of IFN detection in circulating pDCs is explained by the fact that, in vivo, this activation requires stable interactions with infected cells, as shown in vitro. Moreover, studies using the mouse MCMV systemic infection model demonstrated that pDC IFN production (i) occurs specifically in the marginal zone of the spleen in tight contact with infected cells [24, 79, 83] and (ii) is promoted by cell-intrinsic signaling via LFA1 [79] and TNFR1/2 [83].

### During peripheral viral infections, pDCs contribute to IFN-I production in draining LNs

pDCs significantly contribute to IFN production in the draining LNs of mice infected subcutaneously with VSV since IFN-α titers in lymph node homogenates were significantly decreased upon in vivo administration of an anti-PDCA1 antibody [105]. In modified Vaccinia Ankara (MVA)-infected mice, indirect evidence supported IFN-I production by pDCs [106]. Hence, during peripheral infections, pDCs contribute to IFN-I production in the LNs draining the sites of viral inoculation in vivo (Table 4).

### During peripheral viral infections, the contribution of pDCs to IFN-I production in barrier tissues is context-dependent

In the eye, pDCs reside in the anterior stroma of the cornea, where they make a major contribution to IFN-I production in response to a local infection with HSV-1, as demonstrated by the strong reduction in this response upon local interference with pDC functions (Table 4) [108]. To locally deplete pDCs or inhibit their IFN production, the researchers of a previous study performed subconjunctival injections of diphtheria toxin or of a TLR9 antagonist in the eyes of BDCA2-hDTR mice [108].

In the lung, during respiratory viral infections, contrasting results have been obtained for IFN-I production by pDCs (Table 4). They were reported to produce IFN-I/IIIs upon intranasal infection with IAV, as assessed by measuring IFN-α/β titers in lung homogenates of mice treated or not treated with an anti-Bst2 antibody [109, 111] or by using IFN-λ reporter mice [113]. Lung pDCs also produce IFN-I during murine infection with SARS-CoV-1 [118] but not during infection with Newcastle disease virus (NDV) [96] or respiratory syncytial virus (RSV) [109]. Lung pDCs do not produce IFN-λ during NDV infection [113]. A sensitivity threshold could be ruled out in some of these studies, as other cells were found to readily express IFNs in the same tissue, for example, alveolar macrophages for IFN-α production or epithelial cells for IFN-λ in the case of NDV infection [96, 113], and epithelial cells for IFN-α in the case of RSV and IAV, as assessed by in situ hybridization with a riboprobe specific for IFN-α4 [109].

In the skin, pDCs are very scarce under homeostatic conditions but are recruited upon the recognition of inflammatory stimuli. The percentages of pDCs were significantly increased in skin biopsies of patients infected with viruses, such as Molluscum contagiosum virus (MCV) [121], human herpesvirus type 7 (HHV7) [127], and viral warts [128]. Specifically, pDCs were absent from uninflamed skin lesions induced by human papillomavirus (HPV) but infiltrated their inflamed counterparts, which correlated with local induction of the IFN-induced MXA viral restriction factor [128]. Patients suffering from warts due to *Verruca vulgaris* infection, hypogammaglobulinemia, infections, and myelokathexis (WHIM) syndrome exhibit reduced blood pDC numbers, loss of IFN-α production by mononuclear cells upon stimulation with HSV-1 or CpG-DNA, lack of pDC infiltration and induction of IFN-induced MXA viral restriction factor [129]. This suggested that the susceptibility of WHIM patients to viral warts results from the abnormal homeostasis and functionality of their pDCs. However, it remains to be rigorously established whether pDCs produce IFN-Is in the skin during local viral infections. Addressing this question in mice should be facilitated by the novel SCRIPT reporter mouse model enabling in situ identification of pDCs and their IFN-I expression (Table 2) [24].

In genital lesions induced by human papillomavirus (HPV), an increase in pDCs was reported, and pDCs were activated in vitro by HPV virus-like particles [130]. HPV persistence in women might be associated with low pDC and high regulatory CD4+ T-cell counts [131]. This finding suggests a possible role for pDCs in local IFN-I production and virus control during HPV infection of genital mucosae. However, this hypothesis remains to be formally tested. In the endocervix of SIV-infected macaques, pDCs were recruited very early after virus inoculation and appeared to be a main source of inflammatory cytokines, including IFN-Is (Table 3) [132]. In contrast, in the vaginal/cervical tissues of mice infected intravaginally with HSV-2, pDCs were not found to be a major source of IFN-α (Table 4) [92].

In the gut, pDCs were reported to be activated in several models of enteric viral infections, such as rotavirus (RV) and SIV (Tables 3–4). During RV infection in piglets, intestinal pDCs and, to a lesser extent, cDCs appeared to be the main source of IFN-Is, whereas there was no or minimal contribution from monocytes/macrophages, as assessed ex vivo by flow cytometry [123]. Notably, in RV-infected mice, IFN-λ is mainly produced by intestinal epithelial cells and not by pDCs [113]. During SIV infection, pDCs upregulate beta7 integrin, which promotes their migration from the bloodstream to the gut and gut-draining LNs, where they produce IFN-α and other inflammatory cytokines, including TNF and MIP1α [98, 101–104]. pDC accumulation in the infected gut mucosae was also detected in ileal biopsies of HIV-1-infected patients (Table 3) [100].

In summary, only a few studies show a clear and undisputable contribution of pDCs to IFN-I production in barrier tissues during peripheral viral infections. This suggests a major context dependency of this function. The virus and tissue type largely impact this pDC function, and the host species may play a role as well. More studies are needed to understand this complexity and precisely identify the underlying mechanisms controlling pDC activation for IFN-I production in nonlymphoid barrier tissues during peripheral viral infections. Importantly, it might be technically difficult to detect IFN-producing pDCs when their frequency is very low and a limited number of cells are analyzed. This problem of the sensitivity threshold may be able to be overcome in mouse studies by using *Ifnb1*^*Eyfp*^ reporter mice, in which the fluorescent reporter protein EYFP is not secreted and has a long half-life, allowing cumulative detection of all the pDCs that produced IFN at one time point over the >12 hours preceding the analysis (Table 2) [83]. However, even this method might be limiting in some settings. Indeed, pDC IFN production could be clearly revealed in the lungs of mice infected with *Mycobacterium tuberculosis* only by using highly sensitive fluorescent reporter mice, enabling the irreversible tagging of cells at the time of IFN production via a fate mapping strategy [133]. In these mice, transient induction of the Cre recombinase under the control of the *Ifnb1* promoter removed a transcriptional stop cassette in the Rosa26-LSL-Ai16 mutant allele, leading IFN-producing cells to permanently turn on the expression of a fluorescent reporter. As long as they remained alive, they were able to be detected even if they were studied at a later time point than that of their peak IFN-I production. Moreover, this reporter is highly sensitive because the fluorescent signal resulting from IFN-I production is driven by a strong promoter and is not proportional to the level of *Ifnb1* expression in the cells. Thus, this type of approach could allow the analysis of pDC IFN production under conditions where the frequency of pDCs or their activation are especially low, particularly in nonlymphoid tissues and during peripheral infections.

### During viral infections, noninfected pDCs produce IFN-I by sensing engulfed viral nucleic acids via TLR7/9 in vivo

Similar to what was observed in vitro, pDCs appear rather resistant to viral infections in vivo (Table 3). This resistance was proposed to be a result of the high basal expression of ISGs, including IRF7, in resting pDCs. However, in *Ifnar1*-knockout (KO) mice infected with MCMV [21, 79], pDCs were still highly resistant to infection. Moreover, even in pDCs isolated from MCMV-infected *Ifnar1*-KO mice, IFN production still occurred predominantly in noninfected pDCs [79]. Indeed, as shown in vitro, pDC IFN production during viral infections in vivo is driven by TLR7/9-dependent endosomal recognition of engulfed viral nucleic acids. TLR9 is mainly involved in the recognition of DNA viruses, such as MCMV, as confirmed by the impairment of IFN production in MCMV-infected *Tlr9*-KO mice [41, 42, 79, 80, 124]. In fact, TLR7 cooperates with TLR9 for pDC activation during MCMV infection, and only double *Tlr7/Tlr9*-KO mice completely recapitulated the pDC defects and susceptibility observed in *Myd88*-KO mice [124]. TLR7 is required for the sensing of multiple RNA viruses in vivo, including LCMV [88, 134], IAV [43, 135] and SARS-CoV-2 [136]. During NDV infection in wild-type (WT) mice, pDCs resist infection and produce IFN via an endosomal Myd88-dependent pathway. However, in *Ifnar1*-KO mice, pDCs become infected and produce IFN upon endogenous detection of viral infection by cytosolic sensors [95]. Synthetic ligands mimicking microbial ligands of TLR9 and TLR7 have been generated and used to dissect the molecular pathway downstream of Myd88, but these artificial molecules did not exactly reproduce the signaling cascades induced in pDCs during in vivo viral infections [79]. Low amounts of IRF7 were found to be necessary and sufficient to promote IFN production by pDCs via Myd88-dependent pathways [79, 137].

### pDC IFN production is tightly regulated in intensity, time and space during viral infections

pDC IFN-I production has been shown to be tightly regulated in vivo during several viral infections, with a sharp peak confined to less than 24 h, and restricted to a small proportion of the cell population in mice during infections with NDV [96], MCMV [81] or LCMV [88] and in macaques during the acute phase of SIV infection [98]. During MCMV infection, IFN production by splenic pDCs peaks 36-40 h after infection [81], occurring in the marginal zone in contact with infected cells [24, 83], likely stromal cells or marginal zone metallophilic macrophages [138], and with only a very small fraction of pDCs producing IFNs [24, 81, 83]. The spleen marginal zone is characterized by open sinuses where the incoming blood flow is slowed, and the particles it contains, including viruses, can be trapped with great efficiency by marginal zone metallophilic macrophages that are endowed with high phagocytic activity. Upon footpad VSV or MVA infection, pDC IFN production also occurs in a specific microenvironment within the draining LN, namely, in the subcapsular sinus where the afferent lymph enters and where infected subcapsular sinus macrophages monitor/filter the afferent lymph for pathogens and antigens [105, 106]. Thus, both in the spleen and in the LNs, pDC IFN production might be driven by their ability to recognize, and respond to, infection of the macrophage sentinels that filter the incoming body fluids and promote early but contained replication of intracellular pathogens in a manner that promotes protective innate immune responses and the downstream activation of adaptive immunity [139, 140]. However, this hypothesis remains to be formally demonstrated in vivo, and several remaining questions remain to be resolved, including (i) how pDCs discriminate infected macrophage sentinels from their uninfected counterparts in the spleen and LNs, (ii) whether specific endocytic receptors are involved in material transfer from infected macrophages to pDCs, and (iii) whether other microanatomical niches exist in body barrier tissues to promote local pDC IFN production during peripheral viral infections.

The tight spatiotemporal control of pDC IFN-I production is likely critical for preventing the exacerbation of inflammation and the ensuing development of immunopathology or autoimmunity. Indeed, mice deficient in SiglecH, a C-type lectin expressed on pDCs that inhibits IFN production downstream of TLR7/9 recognition [73], exhibit prolonged IFN responses during persistent MCMV infection and develop an IFN-I-dependent severe form of systemic lupus-like autoimmune disease [141]. However, this was not the case when these mice were infected with IAV or LCMV clone 13 [142].

### Mechanisms controlling the spatiotemporal regulation of pDC IFN production during viral infections

If it is true that only specific and limiting microanatomical niches simultaneously provide the appropriate combination of activating signals, then this process would contribute to the tight regulation of pDC IFN production in intensity and space. Indeed, only the fraction of pDCs that access the right niche at the right time would be activated. However, it is probably not the major factor limiting the fraction of pDCs that produce IFNs, because not all pDCs that are in close contact with infected cells produce IFN-I/IIIs, and because pDCs tend to all cluster together at the time of their peak production of IFN-Is [24, 83].

It is possible that pDCs need to not only access positive signals but also escape inhibitory signals. Indeed, pDCs isolated ex vivo from mice infected with HSV-1 or macaques infected with SIV were shown to be in a “refractory” state, preventing them from producing IFN-Is in vitro in response to viral-type stimuli [98, 143]. In vitro studies have shown that human pDCs exposed to free HIV-1 viral particles escape this “refractory” state by maintaining an immature IFN-prone state [144]. However, this might not be the case in vivo, as pDCs isolated from patients undergoing viral rebound upon analytic treatment interruption showed a transient decline in their ability to produce IFN-α in vitro, associated with decreased levels of phosphorylated IRF7 and NF-κB that inversely correlated with plasma IFNα2 levels, suggesting that pDCs were refractory to in vitro stimulation after IFN-α production in vivo [99]. It is possible that the collective migratory behavior of pDCs toward infected cells in the spleen of infected mice and their tight clustering [24, 83] promotes a quorum sensing mechanism, whereby the pDCs that first produce IFNs transmit inhibitory signals acting locally on their neighboring pDCs tightly packed in the same cellular clusters to prevent overshooting of the response and its subsequent deleterious consequences, such as a greater risk of developing autoimmunity, inflammatory diseases or immunopathology. Another possibility is the existence of negative feedback signaling. Autocrine or paracrine responses in pDCs can terminate IFN production, for example, via the induction of inhibitory MER tyrosine kinases [145]. pDCs can sense the IFN-I produced by other cells to terminate their IFN production when they reach a given threshold, a mechanism supported by the inhibition of human pDCs upon engagement of their LILRA4 inhibitory receptor by the Tetherin ligand induced by IFN-Is [146]. Once exposed to persistent chronic infection by LCMV, splenic pDCs enter a prolonged refractory state, making them hyporesponsive when exposed to other viral challenges in vivo, such as MCMV [89]. This process has been termed “pDC exhaustion” and is self-maintained both by the proliferation of splenic pDCs and by increased input from altered bone marrow pDC precursors already programmed for IFN-I production inhibition [134]. A similar process was reported in macaques infected with SIV [98].

Several mechanisms could explain the context-dependent contribution of pDCs to IFN-I production in barrier tissues during peripheral viral infections. Since pDCs are likely activated in vivo by infected cells rather than free viral particles, different viruses may not induce comparable contributions of pDCs to local IFN production in the same tissue because of differences in their cellular tropism or in their manipulation of the expression of adhesion molecules or danger signals in the same target cells. In addition, several microenvironmental factors, including signals from the microbiota [147–150], the cytokine milieu [151], and, putatively, the nature of the cellular interactions established locally by pDCs, can differentially shape the ability of pDCs to produce IFNs across tissues. Future studies using reporter mice enabling the specific identification of pDCs in tissues and tracking of IFN production (Table 2) combined with spatial transcriptomics, multiplex confocal microscopy and pharmacological perturbations should shed new light on the cellular and molecular mechanisms controlling the spatiotemporal regulation of pDC IFN production during viral infections.

### pDCs are seldom the exclusive source of IFN-Is during viral infections

During systemic MCMV infection, low IFN levels are produced by cells other than pDCs, including stromal cells [152, 153], via mechanisms independent of the TLR7/9-to-Myd88-to-Irf7 and TLR3-to-TRIF-to-Irf3 signaling pathways [80, 82, 154]. However, IFN production by other cells usually requires STING-dependent cytosolic sensing of viral nucleic acids in infected cells [153]. Approximately 24 h after systemic LCMV infection, splenic pDCs contribute to IFN production [84, 85, 88], but the majority of these cytokines are produced between 48 and 72 h after infection; this process is preserved in the face of pDC depletion [16, 71, 87, 155] and requires MAVS and MDA5 but not TLR/Myd88 signaling [86, 87], indicating that it originated from cells other than pDCs and likely encompass cDCs [87].

During footpad infection with VSV, in the draining LNs, both pDCs and infected subcapsular sinus macrophages were found to be critical contributors to IFN-I production (Table 4). Both populations produced similar levels of cytokines ex vivo upon isolation from infected animals, and their individual depletion in vivo significantly decreased IFN-I titers in lymph node homogenates [105]. In footpad infection of mice with MVA, pDC depletion did not reduce IFN-α titers in lymph node homogenates [106].

Only a few studies have examined the contribution of pDCs to local IFN-I production in nonlymphoid barrier tissues upon peripheral viral infection. In a mouse model of ocular infection by HSV-1, local depletion of corneal pDCs or selective inhibition of their TLR9 responsiveness strongly reduced IFN-I production (Table 4) [108]. During intranasal infection of 129S7 mice with IAV, administration of an anti-Bst2 antibody dramatically reduced IFN-α titers in bronchoalveolar lavage fluid, suggesting that pDCs play a major role in local IFN-I production in infected lungs (Table 4) [111]. In contrast, in BDCA2-hDTR mice infected intravaginally with HSV-2, pDC depletion upon diphtheria toxin treatment did not decrease IFN-α titers in homogenates from vaginal/cervical tissues (Table 4) [92].

Hence, pDCs are a major source of IFNs in the spleen during many systemic viral infections and in the draining LNs during peripheral infections, but they are seldom the exclusive source of these cytokines. This is also likely the case in peripheral tissues during local infections, although further studies are necessary to assess this phenomenon. This raises the question of the physiological role of pDC IFN production compared to that of IFNs produced by other cells.

During intranasal NDV infection, whereas only alveolar macrophages produced IFN-Is in control conditions, their depletion allowed systemic viral spreading, leading to the activation of splenic pDCs for IFN-I production (Table 4) [95]. This suggested that pDC IFN-I production might act as a failsafe mechanism mobilized mainly in secondary lymphoid organs when viruses escape local immune control at the site of viral entry [156]. In this pathophysiological context, the benefits for the host of high-level production of circulating IFNs by pDCs might supersede the deleterious effects that this could cause on certain cell types or tissues [1]. In this case, pDC IFN production might be required to safeguard the whole organism against further virus spread and its pathological consequences by potentially reinforcing cell-intrinsic antiviral immunity in all of the host cells [1]. We will next discuss whether this hypothesis is supported by experimental data and, more generally, whether pDC responses are beneficial, dispensable or deleterious for the infected host.

## Are pDC responses beneficial, dispensable or deleterious for the infected host?

### Only a few animal infection models have documented a critical contribution of pDCs to viral control and overall host resistance, and pDC IFN-I production appears to be largely redundant in human antiviral immunity

In a mouse model of corneal HSV-1 infection, local interference with pDC IFN production led to increased keratitis and nerve loss, as well as increased viral titers, both in the cornea and its draining LN, ultimately accelerating host death [108]. In systemic infection of mice with HSV-2, at intermediate doses of virus inoculum (10^5^ pfu/mouse), a strong and significant increase in mortality was observed in animals specifically depleted of pDCs [92]. Hence, pDCs and IFN-I production appear to be critical for mouse resistance to HSV-1-induced keratitis and systemic HSV-2 infection (Tables 3–4).

In a mouse model of footpad VSV infection, pDC depletion promoted VSV propagation from the popliteal to the inguinal LN, showing that pDCs contributed locally to viral control (Table 4) [106].

In 129 Sv mice infected with MHV, anti-Bst2 antibody treatment [93] or *Tcf4* haplodeficiency [90] decreased circulating IFN-I levels, increased viral titers, and led to increased liver damage, as assessed by measuring the serum levels of alanine 2-oxoglutarate-aminotransferase (ALT). These results suggested a critical role for pDCs and IFN-I production in viral control and limiting morbidity. However, these effects were much weaker than those observed in *Ifnar1*-KO mice, showing that pDCs were not the exclusive source of the IFNs required for complete host protection [93]. Moreover, anti-Bst2 antibody treatment or *Tcf4* haplodeficiency affected not only pDCs but also other cells, including tDCs [65, 157], which could confound the interpretation of the role of pDCs when using these experimental approaches. Diphtheria toxin administration to BDCA2-hDTR C57BL/6 mice specifically depleted pDCs, which led to increased wasting and mortality; although this effect was considerably weaker than that in *Ifnar1*-KO mice. The serum alanine transaminase (ALT) levels and viral loads were also elevated [65]. In contrast, diphtheria toxin administration to CX3CR1^DTR^ C57BL/6 mice also depleted tDCs in addition to pDCs, which completely rescued the animals from MHV-induced death, consistent with decreased wasting, ALT levels and viral loads compared to those in animals depleted of pDCs only. Hence, this comparison revealed a deleterious role of tDCs during the MHV infection of mice depleted of pDCs, whose underlying mechanism was dependent on tDC IL-1β production. Indeed, IL-1β neutralization in mice depleted of pDCs was sufficient to significantly decrease wasting, ALT levels and viral titers [65]. Taken together, these results showed that (i) the increased pathology observed in mice specifically depleted of pDCs was largely due to a dysregulated tDC/IL-1β response rather than to an impaired IFN-I response, (ii) pDCs are dispensable for host resistance to MHV infection in the absence of tDCs, and (iii) IFN-I-dependent host resistance to MHV infection in C57BL/6 mice was largely independent of pDCs (Table 3). Hence, these findings call into question previous interpretations on the critical role of pDC IFN-I production during MHV infection and, more generally, infections by other coronaviruses [93], as discussed later in this review.

In other animal models of viral infections in vivo, pDC depletion or functional impairment did not strongly compromise viral control or increase host morbidity/mortality, as illustrated by the nonexhaustive examples discussed below (Tables 3–4). During systemic LCMV infection, pDCs are dispensable for viral control [90]. During systemic MCMV or VSV infection, pDC depletion only slightly and transiently enhanced viral replication [70]. During intravaginal infection with HSV-2, pDC depletion using anti-Bst2 antibodies enhanced infection-induced mortality, especially during prophylactic treatment of animals with CpG [158, 159]. However, in this model, other hematopoietic and nonhematopoietic cells were required for CpG-dependent survival [159], and they might be directly affected by anti-Bst2 treatment, thus confounding the interpretation of the role of pDCs. In BDCA2-hDTR mice infected with the same HSV-2 strain and dose, pDC depletion upon diphtheria toxin treatment increased neither viral titers nor mortality [92]. Hence, pDCs are dispensable for viral control and global host resistance to vaginal HSV-2 infection. This is also the case for footpad HSV-1 infection [92]. During footpad infection with ectromelia virus (ECTV), the administration of an anti-Bst2 antibody to deplete pDCs did not significantly increase mouse mortality [107].

No primary immunodeficiency leading to a specific loss of pDCs or of IFN-I production has been detected in humans. Hence, the physiological role of pDCs in human antiviral immunity has been extrapolated from the analysis of patients harboring loss-of-function mutations in genes encoding endosomal TLRs, especially TLR7, or the molecules involved in the downstream signaling cascade leading to IFN-I production, particularly MYD88, IRAK4 and IRF7 (Table 5) [32, 34, 38, 41–43, 59, 79, 124, 136, 160–168]. As expected, blood pDCs isolated from *TLR7*-, *MYD88*-, *IRAK4*- or *IRF7*-deficient patients were defective in IFN production when exposed in vitro to viral or synthetic TLR7/9 ligands [34, 44, 136, 161, 169]. However, unlike patients with impaired IFNAR signaling [2, 170], patients who are genetically impaired in IFN-I production downstream of TLR7/8/9 signaling do not appear to suffer from life-threatening viral infections [44, 161, 169], except for respiratory IAV and SARS-CoV-2 infections [34, 136, 160, 162, 166, 167]. These observations show that pDCs are not the primary line of defense against most acute viral infections in modern humans under current hygiene and healthcare conditions. Even in the case of respiratory IAV and SARS-CoV-2 infections, it is not clear whether the enhanced susceptibility of patients bearing primary immune deficiencies affecting IFN-I production downstream of endosomal TLR signaling is due to defects in pDC responses or other cells. Indeed, human blood monocytes produce IFN-Is in response to TLR7/8 triggering at much lower levels than pDCs [136], but that could still likely be biologically important. Mining public human and mouse scRNA-seq atlases has shown that in the lungs, TLR7 and IRF7 are coexpressed in monocytes and macrophages, which are much more abundant than pDCs.

**Table 5:** Human genetic polymorphisms and murine mutations associated with defective pDC IFN production

	Human		Mouse	
Gene	Genetic polymorphism	Impact on human pDC function	Mutation	Impact on mouse pDC function
*TLR7*	X-linked recessive *TLR7* variants [136]	Defective IFN production upon in vitro stimulation of blood pDCs with SARS-CoV-2 [136]	*Tlr7*-KO [43]	Defective IFN-I production in vitro and in vivo during viral infections [43, 124]
*TLR9*	N.D.	N.D.	*Tlr9*-KO [38]	Defective IFN-I production during viral infections [41, 42, 124]
*IRF7*	Heterozygous [34] or homozygous recessive *IRF7* variants [166, 167]	Defective production of IFN when pDCs are stimulated in vitro with IAV or SARS-CoV-2 viruses[34], or with TLR7/9 ligands [167]	*Irf7*-KO [32]	In vitro and in vivo inability to produce IFN-Is when exposed to viral or synthetic TLR7/9 ligands [32, 79]
*MYD88*	Homozygous recessive *MYD88* variants [161]	No direct test in vitro of patient pDCs, but only a slight reduction in ISG expression in whole blood samples isolated from SARS-CoV-2-infected patients [160]	*Myd88*-KO [163]	Defective IFN-I production during viral infections [41, 42, 124]
*UNC93B1*	Homozygous recessive *UNC93B1* variants [59]	Defective IFN production upon in vitro stimulation of blood pDCs with SARS-CoV-2 [59]	*Unc93b1 3d* [165]	N.D.
*IRAK4*	Homozygous recessive *IRAK4* variants [161] [160]	Defective IFN production upon in vitro stimulation of blood pDCs with SARS-CoV-2 [59]	N.D.	N.D.
*IFNAR1*	Homozygous recessive *IFNAR1* variants [162]	No direct test in vitro of patient pDCs, but a drastic reduction in ISG expression in fibroblasts transfected with mutated isoforms of IFNAR1 [162]	*Ifnar1*-KO [164]	Defective IFN production when exposed to synthetic TLR7/9 ligands, normal production during viral infections [79]

In summary, caution should be taken when interpreting data from mouse models or analyzing data from patients affected by primary immunodeficiencies affecting other cell types in addition to pDCs. Taking this confounding factor into consideration, detailed analysis of published experimental data in mouse viral infection models or in human patients identified very few cases where pDCs are critical for viral control and host resistance to acute primary infections. This calls into question the dogma that pDC professional IFN-I/III production during infections benefits the host by directly contributing to the control of viral replication through reinforcing intrinsic immunity, which needs to be carefully evaluated by using experimental strategies specifically affecting pDCs and specific readouts beyond the measurement of IFN-I/III production.

### The generally dispensable role of pDCs in host resistance to primary acute infections can be explained by redundancies ensuring the robustness of immune responses

During systemic MCMV infection, *MyD88*-deficient mice were more resistant to viral infection than *Ifnar1*-KO mice, showing that other cell types contributed to IFN-I-dependent antiviral defense in the absence of pDCs [82], consistent with the STING-dependent contribution of stromal cells to this function [152, 153]. Moreover, efficient recognition and killing of infected cells by NK cells could compensate for *Myd88* deficiency but not *Ifnar1* deficiency [82], consistent with enhanced susceptibility to viral infections in patients harboring NK cell defects [171].

During MHV infection, since *Ifnar1*-KO mice are much more susceptible than mice depleted of pDCs [65, 93], some level of redundancy must also exist between different cell types for the production of protective IFN-Is.

During footpad infection with ectromelia virus, the administration of an anti-Bst2 antibody to deplete pDCs in *Batf3*-KO mice constitutively lacking cDC1s was sufficient to induce a dramatic increase in infection-induced death, whereas neither of these two deficiencies alone significantly increased mouse mortality, supporting redundancies between pDC and cDC1 functions to control this viral infection in vivo [107].

These results illustrate how the robustness of host immune defenses against viral infections or other threats is ensured by redundancies and complementarities between molecular sensors and cell types for mounting IFN-I/III, IFN-γ, and cytotoxic cellular immune responses, ensuring efficient induction of these essential antiviral functions in the face of host mutations or viral escape mechanisms that might compromise any, but usually not all, of these redundant/complementary pathways (Tables 3–4) [82].

### pDC responses may even be deleterious in certain viral infections

#### The potential deleterious role of pDCs during SIV and HIV-1 infections

IFN-Is and pDCs appear to play dual roles in the pathophysiology of SIV and HIV-1 infection, as we reviewed previously [1]. A strong and transient production of IFN-Is early after infection or after viral reactivation in patients upon analytic treatment interruption [99] likely benefits the host by lowering the viral set point. Sustained production of low levels of IFN-Is during chronic infection contributes to immune dysregulation and CD4+ T-cell depletion. As a proof-of-concept, in the pathogenic rhesus macaque model of SIV infection, early after virus inoculation, injection of a high dose of IFN-I was protective, while neutralization of endogenous IFN-Is was deleterious, and prolonged IFN-I administration worsened disease in the chronic infection phase [172]. Several studies support the notion that pDC activation can play a deleterious role during HIV-1 infection. IFN-I-induced TRAIL expression on pDCs license them to kill CD4 T cells irrespective of their infection status [173, 174]. Compared with men, women with similar viral loads experienced faster HIV-1 disease progression, which may result in part from the greater responsiveness of women’s pDCs to viral-type stimuli, including HIV-1 [175]. pDC recruitment and activation in the vaginal mucosa of female macaques early after local SIV inoculation contribute to attracting and activating CD4+ T cells, which can then be infected and promote virus dissemination from the site of entry [132].

#### Context-dependent beneficial, dispensable or deleterious roles of pDCs in respiratory viral infections

##### PMV infection

Pneumonia virus of mice (PMV) is a natural rodent pathogen that mimics RSV infection in human infants. Transient pDC depletion during primary infection with PMV in BDCA2-hDTR neonates decreased IFN-I titers, increased viral load, promoted severe bronchiolitis, and predisposed animals to asthma development upon reinfection in adulthood (Table 4) [120]. These results show that pDCs are beneficial in this viral infection model.

##### Influenza A virus (IAV) infection

Both IFN-Is and IFN-IIIs contribute to resistance to IAV infection in mice. At low viral loads, IFN-IIIs appear to be critical and sufficient for viral replication within the lung, whereas at high viral loads, both IFN-Is and IFN-IIIs are required to limit IAV dissemination [176]. However, depending on both the mouse genetic background and virus strains, unbridled IFN production can occur, which is detrimental to the host because it can fuel excessive inflammation in the lungs, causing severe immunopathology [111, 176, 177]. This explains why contrasting effects, ranging from beneficial to dispensable or even detrimental, have been reported for pDCs in murine models of IAV infection (Table 4).

In 129 mice infected with the X31 IAV strain, IFN production was high and detrimental. Depletion of Bst2^+^ cells or administration of TLR7 antagonists decreased lung immunopathology and morbidity, suggesting that pDCs play a deleterious role in this infection model [111, 116].

In C57BL/6 mice infected with the X31 IAV strain, pDC depletion upon anti-Bst2 antibody administration did not alter morbidity, but it decreased the production of anti-IAV antibodies [114]. In C57BL/6 mice infected with the A/PR/8/34 IAV strain, the constitutive absence of pDCs in mice bearing a hypomorphic mutation of *Ikaros* did not alter viral titers, morbidity or neutralizing antibody titers, although it delayed CD8+ T-cell recruitment [110]. These results show that pDCs are dispensable for the control of infection with two different IAV strains in C57BL/6 mice.

In BALB/c mice infected with the A/PR/8/34 IAV strain, pDC depletion upon anti-Bst2 antibody administration decreased lung IFN-α titers, transiently reduced lung virus burden, slightly delayed weight loss, and increased the recruitment and activation of DCs and monocytes but did not ultimately prevent fatal outcomes compared to WT controls [112]. These results suggested that pDCs had a suppressive effect on the pulmonary inflammatory response of other mononuclear phagocytes to IAV infection in BALB/c mice but contributed to lung inflammation, resulting in a neutral net effect of pDC depletion on disease outcome. In BALB/c mice infected with the A/JAPAN/305/57 IAV strain, genetic inactivation of *Fasl* delayed mortality, lung-infiltrating pDCs expressed FasL and were able to kill antiviral CD8+ T cells in vitro, and adoptive transfer of WT but not FasL-KO pDCs into *Fasl*-KO mice decreased antiviral CD8+ T-cell responses and accelerated mortality [115]. These results thus suggested that pDCs can dampen host resistance to IAV infection by eliminating antiviral CD8+ T cells.

Most laboratory mouse strains bear nonfunctional alleles of *Mx1*, an ISG encoding the main restriction factor against IAV [117, 178]. These mice are thus deficient in Mx1-mediated intrinsic antiviral immunity against IAV infection. This could explain at least in part the contrasting effects of IFN-I observed in certain combinations of mouse genetic background and virus strains, which in turn could profoundly alter the antiviral functions of pDCs. One study attempted to investigate this issue by generating C57BL/6 mice congenic for functional *Mx1* alleles but deficient in *Myd88* or *Tlr7* or depleted of pDCs upon anti-Bst2 antibody administration. After infection with the SC35M IAV strain, all of these mice exhibited increased viral lung titers. *Myd88*-KO and *Tlr7*-KO mice also exhibited a strong increase in infection-induced death. However, the impact of pDC depletion on mortality has not been reported [117]. These results show that Myd88 and Tlr7 responses are critical for resistance to IAV infection in these experimental settings and that Bst2^+^ cells contribute to this protection, suggesting a possible beneficial role for pDCs.

In all of the above experiments, caution should be taken before making any definite conclusions about the role of pDCs since the administration of anti-Bst2 antibodies, TLR7 antagonists, or *Myd88*/*Tlr7* genetic deficiencies impact other cells in addition to pDCs, including activated monocytes and B cells, macrophages, and tDCs that are also recruited to the lungs during IAV infection [157] (Table 1). Further studies using tools specifically targeting pDCs are thus required to rigorously determine their role in mouse models of IAV infection.

##### Respiratory coronavirus infections

Highly pathogenic coronaviruses, such as MERS-CoV or SARS-CoV, have emerged during the last decade, leading to the worldwide COVID-19 pandemic. Severe COVID-19 appears to be largely due to inadequate or excessive host immune responses. Complete loss of IFN-I responses compromises host antiviral defenses and is a major factor that occurs in approximately 20% of severe COVID-19 cases [136, 160, 162, 166, 167, 179, 180]. In a longitudinal study comparing cytokine levels in the peripheral blood with viral loads in nasopharyngeal swabs, the viral infection of patients suffering from moderate COVID-19 was effectively controlled within a week via early and strong but transient IFN production, while the viral infection was unable to be controlled in severe COVID-19 patients, who also displayed sustained production of IFNs, as well as proinflammatory cytokines [181]. This suggested that, depending on its timing and magnitude, IFN production in the airways could exert opposite effects on host resistance to pulmonary viral infections. Indeed, a rapid, strong but transient IFN-I/III response in the upper airways contributes to early viral control and protection [182]. On the other hand, the inability to rapidly control viral replication leads to delayed but sustained IFN-I induction, fueling persistent inflammation and immunopathology in the lung. This hypothesis is true in murine models of MERS-CoV and SARS-CoV-1 infections, where an early IFN response is crucial for controlling viral dissemination, whereas a late response becomes deleterious, promoting exacerbated inflammatory responses in myeloid immune cells that cause pulmonary immunopathology [118, 119].

pDCs detect coronaviruses via TLR7 and were found to be the main source of IFNs among human peripheral blood mononuclear cells exposed to MERS-CoV or SARS-CoV-2 in vitro [48, 57, 59, 136, 183]. pDCs are optimally activated by cells infected with SARS-CoV-2 [48, 183], and NRP1-dependent mechanisms regulate their activation [184, 185]. Patients suffering from severe COVID-19 exhibit a strong decrease in the number of circulating pDCs and a defect in their ability to produce IFN-I in vitro upon exposure to SARS-CoV-2 [48, 59, 136, 186]. This was interpreted as proof of defective pDC-dependent control of viral infection in these patients, which could be responsible for their life-threatening susceptibility to COVID-19. Hence, it has been proposed that pDCs play a major protective role against human SARS-CoV-2 infection [48, 59, 136, 167, 183, 186]. However, akin to what has been shown in HIV-1 and SIV infections [98, 99], the decrease in circulating pDCs observed in patients with severe COVID-19 could result from their recruitment into the infected lung [187], and the failure of circulating pDCs to produce IFN-I upon in vitro stimulation could be due to the refractory state induced by the prior in vivo production of cytokines by pDCs [188]. This alternative interpretation of the observations from patients with severe COVID-19 raises the hypothesis that pDC IFN production in the lungs during SARS-CoV-2 infection could exert a detrimental effect by promoting the dysregulated activation of monocytes and macrophages directly responsible for the cytokine storm causing severe COVID-19 [181, 189–191]. This hypothesis is consistent with observations in an animal model of SARS-CoV-infected BALB/c mice in which, at 1 dpi, lung SiglecH^+^ cells harboring pDCs expressed higher levels of *Ifnb1* and *Ifna4* mRNA than did SiglecH-negative cells and alveolar macrophages, and treatment with depleting anti-CCR2 or anti-BST2 antibodies strongly decreased the number of lung monocytes and pDCs, protected against mortality, and reduced alveolar edema, bronchial epithelial sloughing, and vascular leakage while decreasing viral titers only ~2-fold (Table 4). Monocytes are a major source of TNF, and neutralization of TNF decreases disease severity [118]. It is possible that the lung harbors a particular susceptibility to the detrimental consequences of excessive production of IFNs by pDCs compared to other tissues, since lung pDC IFN-I production was recently shown to drive severe disease in mice susceptible to *Mycobacterium tuberculosis* infection [133]. To rigorously dissect the role of pDCs in respiratory coronavirus infections, further kinetic and mechanistic studies are needed in animal models permissive to both SARS-CoV-2 infection and the specific in vivo tracking and targeting of pDCs to compare their activation in the blood versus that in the respiratory apparatus and to examine the consequences of their depletion or inhibition of IFN production on viral loads, morbidity, mortality and the accumulation of dysfunctional, deleterious, inflammatory mononuclear phagocytes in the lungs.

## How are pDCs shaping host responses to viral infections?

### Strong evidence that supports the prevailing dogma that pDC IFN production is crucial for antiviral defense by boosting intrinsic antiviral immunity is lacking

During systemic MCMV infection, neither *Myd88* deficiency nor pDC depletion affected the induction of ISG expression in the spleen, which is consistent with the greater resistance of *Myd88*-KO mice to infection than of *Ifnar1*-KO mice, thus indicating that efficient cell-intrinsic antiviral immunity is achieved even in the absence of pDCs or IFNs (Table 3) [82]. In many other studies, it is not possible to rigorously determine whether/to what extent pDCs contribute to boosting intrinsic antiviral immunity because the experimental designs used showed off-target effects and lacked analyses of intrinsic immune responses downstream of IFN-I/III production and the inclusion of *Ifnar1*-KO control animals. Hence, future studies are needed to answer that question clearly, combining the use of mutant mouse models enabling specific, efficient, and sustained targeting of pDCs, with more complex readouts moving beyond the mere measurement of IFN production and viral titers, toward comparative quantitative measurement of IFN-I responses (e.g., ISG induction, morbidity, and mortality) between WT mice, animals deficient in pDC IFN-I production and *Ifnar1*-KO mice.

### Role in the activation of innate antiviral immunity

Upon viral sensing, in addition to secreting IFNs, activated pDCs produce a large repertoire of inflammatory cytokines, such as TNF, IL-18, and IL-6, and chemokines, such as MIP-1α and MIP-1β [21, 31, 41, 79, 81, 83, 92]. IL-12 is also produced by mouse pDCs but not by human pDCs [31, 71, 81]. These soluble factors allow pDCs to recruit and activate multiple innate and adaptive immune cells through direct and indirect mechanisms [1, 62, 64]. Moreover, pDCs can also modulate immune responses by establishing contact-dependent interactions with immune cells. In the next section, we will focus on NK cells, cDC1s, and T and B lymphocytes as prominent examples of pDC-dependent activation of innate and adaptive immunity, respectively (Tables 3–4).

#### Role in NK cell activation

The coculture of pDCs and NK cells isolated from human peripheral blood promoted the cytotoxicity and IFN-γ production of NK cells [192]. The cytokines released by pDCs exposed to viral or synthetic TLR7/9 ligands were crucial for this crosstalk, with IFNs promoting cytotoxicity and IL-12/IL-18 inducing IFN-γ secretion. Cell-cell contacts may also be involved [193, 194]. In mice deficient in *TLR7/9* or *Myd88* or depleted of pDCs and infected with viruses, such as MCMV, HSV or DENV, NK cell cytotoxicity, and cytokine production were decreased or even abolished [21, 41, 70, 82, 92, 195], thus supporting the contribution of pDCs to NK cell activation in vivo during viral infections.

The specific contribution of IFNs to NK cell activation was addressed in chimeric mice displaying NK cell-autonomous defects in *Ifnar1* [196]. This study revealed that NK cell cytotoxicity and cytokine production were marginally affected in the absence of cell-intrinsic IFN responses. Rather, NK cell proliferation is strongly dependent on IL-15 trans-presentation by DCs, which is itself induced by cell-intrinsic responses to IFNs [196]. Thus, these results confirmed and extended previous studies on the role of different innate cytokines in NK cell activation [197] by showing that, during viral infections, IFNs produced by pDCs and other cells indirectly promote NK cell proliferation via the licensing of cDC1s for IL-15 trans-presentation [198].

In contrast, IL-12 and IL-18 have direct effects on IFN-γ production by NK cells in a tissue-dependent manner [199]. pDCs are not the exclusive source of these cytokines. Indeed, in MCMV-infected pDC-depleted mice, NK cells produce fewer IFN-γ at early time points after infection, while later, this effect is actually enhanced [70]. This was due to the heightened production of IL-12 by cellular sources other than pDCs, consecutive to the removal of the break imposed by pDC IFNs [41, 71]. The IL-18 produced by activated pDCs also promoted IFN-γ production by NK cells, as shown in HSV-1-infected *IL-18R*-deficient mice [200].

#### Role in cDC1 activation

In MCMV-infected mice or upon TLR7/9 ligand stimulation, loss of the IFN response or pDC depletion transiently hampered the expression of MHC class I or CD86 on cDCs, especially cDC1s [21, 69, 70]. These findings indicate that although IFN responsiveness is essential for the activation of cDCs [21, 79, 196, 201], there is a redundancy of IFN sources promoting this function [70, 82]. However, among IFN-producing cells, pDCs can excel in their ability to release IFNs at the right time and place. pDCs can ensure this spatiotemporal regulation, as they can migrate in response to several chemokines and are highly mobile, especially after viral infections [24, 83, 106]. Indeed, chemokine-dependent guidance attracted pDCs to virally infected cells or T-cell-enriched areas [24, 83, 106], close to cDC1s [198], with which pDCs can eventually interact [106]. Moreover, activated pDCs also produce XCL1 [24, 83], a chemokine prominently released by cytotoxic lymphocytes that is essential for the recruitment of cDC1s, which specifically express its cognate receptor, XCR1. Thus, early during viral infections, via their production of IFNs and XCL1, pDCs might contribute to guiding cDC1 migration toward infected tissue areas, inducing their optimal location and activation. This would in turn promote NK cell responses as well as the uptake and cross-presentation of viral antigens by cDC1s for later induction of antiviral CD8+ T-cell responses upon further migration into T-cell-rich areas [198]. Whether contact-dependent interactions are also required for the cross-talk between pDCs and cDC1s requires future investigations.

### Role in the activation of antiviral effector adaptive immune responses to primary acute infections

During viral infections, the cytokines produced by pDCs can also promote the functional polarization of CD8+ T lymphocytes to effector cytotoxic cells or that of CD4+ T lymphocytes to helper or regulatory cells. pDC IFNs can also stimulate B cells for the production of virus-specific antibodies. Moreover, at later phases after TLR7/9-dependent activation, pDCs can acquire antigen-presenting cell (APC) properties [21, 24, 83], suggesting that they might directly engage in cognate interactions with antiviral T cells. These distinct functions of pDCs are described in detail below (Tables 3–4).

#### Role in CD8+ T-cell activation

During viral infections, pDCs can modulate the survival and expansion of virus-specific CD8+ T cells. The generation of antiviral CD8+ T cells was affected during HSV-1 infection by conditional pDC depletion, as achieved upon diphtheria toxin treatment of *Siglech*-hDTR mice, and during chronic LCMV infection in mice constitutively devoid of pDCs (CKO mice) [69, 90]. During intranasal IAV infection, the recruitment of CD8+ T cells was also delayed in pDC-deficient *Ikaros*^L/L^ hypomorphic mice [110]. Notably, in all these experimental settings, the phenotype observed might not be due only to pDC deficiency, as other cells are also affected, particularly tDCs in *Siglech*-hDTR and CKO mice (Table 1). Indeed, in MCMV-infected mice, CD8+ T-cell numbers are severely reduced only in the absence of both NK cell-dependent and Myd88-dependent responses, supporting the redundancy between several defense mechanisms for promoting protective antiviral CD8+ T-cell responses and, more broadly, host resistance to infection [82]. However, in VSV-infected BDCA2-hDTR mice, the specific depletion of pDCs, which was achieved by diphtheria toxin treatment, led to increased apoptosis and reduced accumulation of vesicular stomatitis virus (VSV)-specific CD8+ T cells [70].

pDC ablation can also affect the activation of effector functions, particularly IFN-γ production by CD8+ T cells induced upon HSV-1 infection [69, 92] or chronic LCMV infection [90], whereas pDCs appear to be dispensable for this function in mice infected with other viruses, such as MCMV [70].

These results show that, in certain viral infections, pDCs can contribute to the promotion of antiviral CD8+ T-cell responses in a significant and nonredundant manner. However, the underlying mechanisms remain to be formally identified, including the role of direct IFN-I effects on CD8+ T cells, indirect effects via cDC1 licensing [106], and the contribution of pDCs to viral antigen presentation to T cells.

Whether pDC loss or functional deficiency can hamper adaptive immune memory responses has not been intensively investigated. Addressing that question will require future studies with tools allowing long-term specific depletion of pDCs.

#### Role in CD4+ T-cell activation

The cytokines produced by pDCs can induce distinct patterns of CD4+ T-cell polarization depending on both the type of virus and the site of infection. In corneal HSV-1 infection, IFNs produced by pDCs are essential for limiting the generation of pathogenic Ex-Treg cells that produce IFN-γ [108], whereas in mice exposed to chronic LCMV infection, IFNs contribute to the optimal activation of CD4 T helper cells [90]. During LCMV infection in the pancreas, in the draining LN, pDCs produce immunosuppressive cytokines, including TGF-β, polarizing CD4+ T lymphocytes into regulatory cells that are able to limit CD8+ T-cell effector functions against virally infected cells, thus limiting tissue lesion and diabetes onset [202]. Contact-dependent interactions were also involved in pDC-dependent induction of CD4+ Treg cells. Indeed, during PMV infection in neonates, Semaphorin4 expressed by pDCs interacts with its ligand Neuropilin, which is present on CD4 Tregs, to promote protection against the development of severe viral bronchiolitis and subsequent asthma [120].

Once cytokine production ceases, pDCs upregulate the expression of both MHC and costimulatory molecules and acquire APC features, including the ability to activate T cells [203]. However, the ability of pDCs to process and present antigens has recently been called into question, as it was claimed to be entirely accounted for by tDCs contaminating the pDC population in many experiments [204]. However, we recently proved at the single-cell level that, in vivo during MCMV infection, once IFN production is terminated, true pDCs undergo reprogramming, leading to the convergence of their transcriptome, phenotype, and function toward cDCs while preserving their core pDC molecular identity [83]. These pDCs expressed CCR7 and migrated to the T-cell zone, thus being in a strategic position to regulate T-cell functions. Moreover, they were able to promote antigen-dependent proliferation of CD4 T cells in vitro much more effectively than the other pDC activation states studied [83]. However, their ability to process and present antigens for antiviral T-cell activation in vivo remains to be tested.

#### Role in B-cell activation

The contribution of pDCs to B-cell activation has been addressed only in a few models of viral infections. During enteric RV infection, IFNs produced by intestinal pDCs promote B-cell activation for the production of virus-specific IgG and IgA antibodies that control viral shedding [122, 123]. During intranasal IAV infection, the depletion of BST2^+^ cells, including pDCs, reduces the production of virus-specific antibodies, but the underlying mechanisms remain to be elucidated [114]. pDCs can promote the generation of extrafollicular B-cell structures in a mouse model of systemic lupus erythematosus [205]; however, whether this is also the case during certain viral infections remains to be investigated.

In summary, several studies have reported that pDCs contribute to the induction and functional polarization of protective innate and adaptive immune responses, both during systemic and local viral infections (Tables 3–4). However, many of these experiments were performed under conditions in which not only pDCs but also other cell types, including tDCs, were affected (Table 1). Hence, whether pDCs can efficiently process and present antigens in vivo to activate T cells, and more generally, to what extent pDCs contribute to protective adaptive immunity, remains to be formally established under experimental conditions allowing specific manipulation of pDCs without off-target effects on other cell populations or confounding side effects [206]. This will require the use of new mutant mouse models allowing the specific inactivation of key pDC genes involved in antigen processing and presentation, the formation of immunological synapses between APCs and T cells, or interactions with B cells.

Although the vast majority of studies analyzing pDC functions have focused on their role in IFN production and the induction of protective intrinsic, innate, or adaptive immunity during viral infections, it is important to realize that pDCs are already very active under homeostatic conditions. Indeed, steady-state pDCs share many morphological, ultrastructural, transcriptomic, and metabolic features with plasma cells that continuously secrete antibodies, including a constitutively active unfolded protein response pathway that is critical for their survival. This finding suggested that steady-state pDCs continuously produce and secrete proteins. Considering the energy supply that this constitutive activity state requires to have been maintained throughout the evolution of vertebrates, it is likely that pDCs not only enabled rapid IFN production upon viral infection but also somehow benefited the host at homeostasis, as suggested by several studies discussed in the last section of this review.

## Contribution of pDCs to homeostatic functions at steady state and their deregulation in autoimmune diseases

**Fig. 4:**
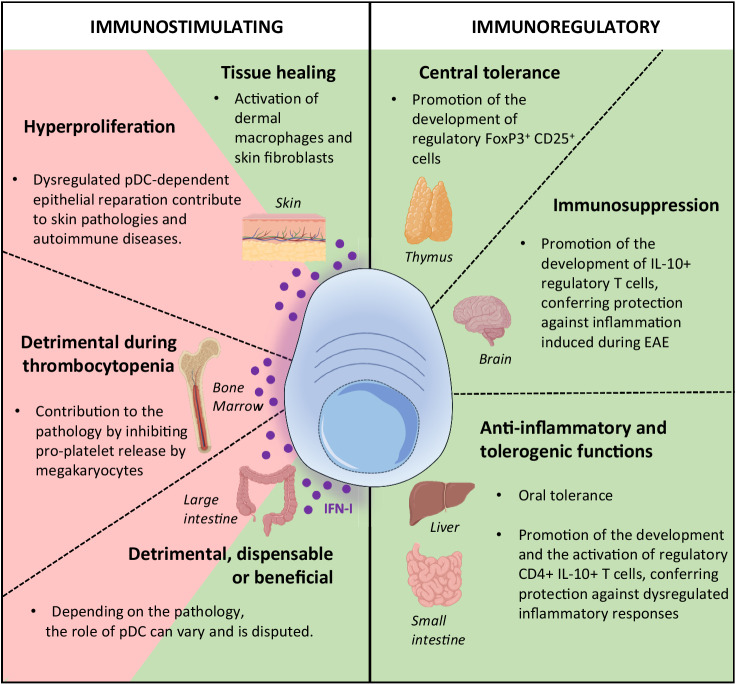
Immunostimulatory vs. immunoregulatory functions of pDCs. In both lymphoid and nonlymphoid organs, pDCs are involved in various biological and pathological processes in addition to their contribution to antiviral defense. pDCs can exert immunostimulating (IFN production) or immunoregulatory (promotion of Treg cells) functions, which can be beneficial (green) or detrimental (red) for the host, depending on the context. For example, pDCs recruited to the skin upon tissue damage promote tissue repair through IFN production, but when dysregulated, this function can be deleterious, promoting skin pathologies and autoimmune diseases. pDCs might be detrimental in bone marrow thrombocytopenia, by inhibiting proplatelet release due to the pathological loss of SiglecH-dependent inhibition of pDC IFN production. How this process might be beneficial and in what pathophysiological context are unknown. The role of pDCs in the large intestine depends on the pathological context and is still controversial. Immunoregulatory pDCs that produce anti-inflammatory cytokines, such as IL-10 or TGF-β, can benefit the host in different contexts. At a steady state, they can promote the expansion of CD4+ Tregs, contributing to central tolerance in the thymus and to oral tolerance in the liver and small intestine. During neuroinflammation, the recruitment of immunoregulatory pDCs to the brain can dampen inflammatory responses and ameliorate tissue lesions

In addition to their contribution to antiviral defense, mainly via their ability to produce IFN-I/IIIs, pDCs are involved in different physiological processes, such as thrombopoiesis and central and peripheral tolerance. However, pDCs have been reported to be detrimental in inflammatory or autoimmune diseases [64], suggesting that perturbations in tissue homeostasis might switch pDC functions from beneficial to detrimental. In the second part of our review, we summarize the main findings regarding the functions of pDCs in homeostasis versus sterile inflammation (Fig. 4).

### Role of thymic pDCs in central and peripheral tolerance

The presence of pDCs in the human and mouse thymus was first reported in 2005 [207]. Thymic pDCs were proposed to induce central tolerance by promoting the generation of FoxP3^+^ CD25^+^ Treg cells, which produce IL-10 and likely act complementarily to cDCs for this purpose [208–210]. In vivo, in a model of graft versus host disease (GVHD) induced in irradiated C57BL/6 recipient mice receiving allogeneic BALB/c T-cell-depleted BM cells, the cotransfer of syngeneic pDCs with allogeneic donor T cells improved survival and prevented GVHD [208]. This protection relied exclusively on CCR9^+^ pDCs, thus indicating a critical role for the responsiveness to CCR9 ligands in regulating pDC trafficking and functions. Indeed, pDC migration into the thymus requires CCR9 and is abrogated when CCR9 expression is downregulated upon pDC activation by TLR7/9 ligands [210]. In the thymus, antigen-loaded pDCs localize to the medulla and induce the clonal deletion of antigen-specific CD4 T cells [210]. These findings suggest that at steady state, pDCs migrate to the thymus and promote central tolerance (Fig. 4), whereas upon sensing microbial dangers, pDCs are redirected to other organs and potential sites of pathogen entry, and switch to an immunostimulatory phenotype. pDCs were also found to likely be involved in peripheral tolerance [211–213], although their ability to directly present antigens to T cells is debated [204, 214].

### Role of peripheral pDCs in systemic autoimmunity

The transcriptional signature of the response to IFNs is very often found in patients suffering from inflammatory disorders or autoimmune diseases, collectively called interferonopathies. Systemic lupus erythematosus (SLE) is the prototypical example of interferonopathy. SLE is characterized by the recognition of endogenous nucleic acids and nuclear antigens by autoantibodies that form immune complexes. In the blood of SLE patients, an IFN-I response signature positively correlates with autoimmunity [215]. As professional IFN producers, pDCs were proposed to be detrimental in SLE [216], although a recent study reported that IFN production by pDCs is impaired in SLE patients and that nonhematopoietic cells are the main source of IFNs [217]. However, the deleterious role of pDCs was supported in two independent models of mice genetically prone to SLE in which introgression of another mutation enabled specific pDC targeting. Specifically, BXSB mice were crossed with BDCA2-hDTR mice to enable conditional pDC depletion upon diphtheria toxin administration [218], and B6.*Sle1. Sle3* mice were crossed with *Tcf4* haplodeficient mice constitutively lacking pDCs [219]. In both models, pDC deficiency significantly reduced tissue damage, consistent with a dampened IFN response signature, autoantibody production, and autoreactive T and B-cell activation.

A key question about interferonopathies is the precise origin and biochemical nature of the self-nucleic acids that trigger pDC activation. Under healthy conditions, the endosomal location of TLR7 and TLR9 prevents pDC detection of their own nucleic acids, and specific nucleases degrade self-nucleic acids from other cells that could be otherwise sensed by pDCs upon extracellular exposure, for example, during cell turn-over or inefficient efferocytosis [220]. This process can be disrupted in patients suffering from interferonopathies, for example, due to loss-of-function mutations of genes encoding nucleases [220], including DNASE1L3 [221], and corresponding mutant mouse models are being used to decipher the mechanisms underlying SLE development. *Dnase1l3*-KO mice develop an SLE-like disease [222], which is abrogated in double-deficient *Dnase1l3*-KO;*Ifnar1*-KO mice [205]. In this SLE model, pDCs promoted disease, including the extrafollicular activation of autoreactive B cells producing anti-DNA antibodies, in a manner dependent primarily on Tlr9 and, to a lesser extent, on Tlr7 [205]. The major types of nucleic acids captured and recognized by pDCs in patients suffering from psoriasis and SLE are complexes composed of self-DNA, the antimicrobial peptide LL37, and high mobility Box 1 (HMGB1), which are released by dying neutrophils during NETosis [216, 223, 224]. This led to a vicious cycle of autoamplification since pDC IFNs, in turn, amplified NETosis in neutrophils [224], and the autoantibodies directed against ribonucleoproteins and LL37/DNA complexes triggered NETosis and B-cell activation, respectively, mainly via FcR-dependent mechanisms [216, 224].

Once activated by TLR7/9 ligands, pDCs trigger an antiapoptotic program, making them resistant to conventional immunosuppressive therapy with glucocorticoids [225]. Hence, targeting pDCs, and specifically their IFN production, provides new treatment options for autoimmune disorders. Recombinant antibodies recognizing pDC receptors, such as BDCA2 or ILT7, could inhibit IFN production by pDCs or deplete pDCs, with promising results both in vitro and in vivo in SLE patients [74, 75, 226, 227]. Synthetic amines able to bind to a minor allosteric pocket of the CXCR4 receptor inhibited IFN production by pDCs in a pristane-induced mouse model of SLE [228] but also exerted anti-inflammatory effects on other cells [229]. Targeting glycolysis and the unfolded protein response (UPR) in pDCs also appears to be a promising therapeutic approach. Indeed, the UPR induces the inositol-requiring enzyme 1α (IRE1α)/X-box binding protein 1 (XBP1)/phosphoglycerate dehydrogenase (PHGDH) axis, which rewires glycolysis to serine synthesis, thus eliminating the tricarboxylic acid (TCA) cycle of pyruvate and inhibiting ATP production required for IFN synthesis [230]. Interestingly, the IRE1α-XBP1–PHGDH axis was inhibited in patients suffering from systemic sclerosis, while pharmacological inhibitors of the TCA dampened IFN production in patient pDCs [230]. Finally, pharmacological treatments inhibiting cytokines other than IFN can hyperactivate pDCs. This is the case for anti-TNF antibodies, which are currently used to treat certain autoimmune diseases, such as rheumatoid arthritis. As TNF modulates both pDC generation and activation, blocking this cytokine leads to pDC hyperactivation [231], which may be involved in the development of lupus-like or psoriasis-like diseases in treated patients. However, during viral infections, cell-intrinsic TNF responses in pDCs early during their activation promote IFN production [83]. Thus, future investigations are required to determine how to reconcile these apparently opposite results.

Box 1 Outstanding questions on pDCs How do pDCs resist viral infection?

- Cell-intrinsic responsiveness to IFNs is not needed.
- Is this resistance linked to the constitutive expression of specific restriction factors?
- Is this resistance linked to specific intracellular routing of incoming viral particles into endolysosomal compartments, preventing fusion and promoting degradation?

What makes pDCs so effective at producing IFN-I in response to viral-type stimuli compared to other immune cells? How do pDCs specifically recognize and respond to infected cells?

- Although TLR7/9 and IRF7 expression are necessary for this function, it is not clear what differentiates pDC responsiveness to this signaling pathway from that of other cells expressing these molecules at similar levels, such as monocytes, macrophages, or cDC2s. For example, neither high IRF7 expression nor IFN-I positive feedback or AP3-driven endosomal routing of TLRs are required for optimal pDC IFN production in vivo during MCMV infection [79]. Thus, other cellular and molecular mechanisms must exist that endow pDCs with a unique ability to sense viral infections by triggering their unique production of all IFN subtypes. It is likely that such mechanisms encompass the early steps of the recruitment of pDCs to microanatomical sites of viral replication in vivo and then of the recognition and engulfment of viral particles or viral material derived from infected cells, which may be unique to pDCs.
- Through which specific receptors do pDCs specifically discriminate infected cells locally from their normal neighboring cells to establish stable contacts only with the former?
- Are these the same receptors enabling pDCs to engulf material derived from infected cells, or does this step involve other receptors?

What are the molecular mechanisms restricting pDC IFN production to only a fraction of these cells?

- Is access to viral-derived nucleic acids a key limiting factor for pDC activation during viral infections?
- Under homeostatic conditions, are some pDCs in a specific transcriptomic or epigenetic state that can activate or inhibit IFN production upon activation due to imprinting by a specific microanatomical environment or past interactions with specific cellular or microbial stimuli?
- To be triggered to produce IFN, do pDCs need to receive several independent specific signals, synchronously or at specific time points relative to one another?
- To be triggered to produce IFN, do pDCs not only need to access positive signals but also need to escape inhibitory signals?
- Is pDC IFN production regulated at the cell population level via quorum sensing?
- Are pDCs able to check their efficacy in inducing an antiviral gene expression program in surrounding cells to stop their IFN production when it is no longer needed? Is the mechanism in mice functionally equivalent to the triggering of the inhibitory LILR4A receptor on human pDCs by the Tetherin induced by IFNs on IFN-responding cells?

What mechanisms are shared versus differing for pDC sensing of virus-infected cells versus of self-DNA/RNA and for the downstream molecular regulation of IFN production?

- In autoimmune diseases, are self-nucleic acids delivered to pDCs by damaged or dying cells through a mechanism resembling the immune synapse observed with infected cells?
- In autoimmune diseases, is there a disruption in some of the mechanisms restricting pDC IFN production during viral infections?

When, where, and how does pDC IFN benefit the host during viral infections?

- What is the role of pDCs in intrinsic antiviral immunity?
- What is the role of pDCs in the activation of innate effector immune cells during acute viral infections?
- What is the role of pDCs in the induction of adaptive immunity
- Are pDCs able to efficiently present antigens to T cells under physiological conditions in vivo?

When, where, and how are pDC responses to viral infections deleterious to the host?

- Are pDCs deleterious when they are activated too late and/or for too long, such that they do not contribute to viral control but fuel deleterious inflammation?
- Are pDCs directly causing damage to infected tissues?
- What is the role of their IFN production versus other functions?

In human patients, is the measurement of blood pDC numbers and of their capacity to produce IFN in vitro after restimulation adequate to understand their activity in vivo in infected tissues?

- A decrease in blood pDCs could correspond to their recruitment into infected tissues.
- The inability of blood pDCs to produce IFN-I in vitro after restimulation could correspond to a refractory state following their prior activation in vivo.
- Hence, caution should be taken when extrapolating the number and activation status of blood pDCs to those of pDCs in lymphoid tissues or infected nonlymphoid tissues.

Can the same pDC switch from immunostimulatory to tolerogenic functions and vice versa?

How are pDC functions shaped by their tissue microenvironment?

Is there a role for pDCs in nonviral microbial infections?

### pDCs in the homeostasis of the digestive tract (liver and gut)

At a steady state, pDCs are present not only in lymphoid organs but also in various nonlymphoid organs, including the gut and liver (Fig. 3) [24, 232, 233]. In the gastrointestinal apparatus, pDCs are scattered in the lamina propria of the villi of the small intestine, whereas they are absent in the large intestine and in enteric organized lymphoid structures, such as Peyer’s patches [24, 232]. In the liver, pDCs constitute an important fraction of CD45^+^ hematopoietic cells [233], but their tissue location has not yet been precisely defined. Hepatic and enteric pDCs were reported to exert anti-inflammatory and tolerogenic effects, specifically by promoting oral tolerance (Fig. 4) [232–235]. Depending on the experimental conditions, hepatic/enteric pDCs can activate CD4^+^ IL-10^+^ Tregs [234, 235] or other tolerance pathways [233]. The IL-27/Ebi3 cytokines might regulate the tolerogenic functions of liver pDCs, particularly their ability to preferentially polarize T cells toward regulatory functions, likely via autocrine signaling [236].

The microbiome can influence both pDC trafficking and function (Fig. 4). pDC trafficking toward both lymphoid and nonlymphoid organs was altered in germ-free mice, especially for CCR2^+^ pDCs, likely because steady-state CCL2 production by monocytes results from their sensing of the microbiota [237]. The sensing by pDCs of commensals or their metabolic products was reported to promote basal tonic production of IFNs, which in turn primed conventional DCs for fast and efficient induction of adaptive immunity to infections [149]. The infant microbiome was also reported to indirectly regulate pDCs [150]. Indeed, upon exposure to a maternal high-fiber diet, the milk microbiome releases propionate, which promotes the transient expression of FLT3L in neonatal gut epithelial cells. This neonatal FLT3L promoted the generation of tolerogenic pDCs that activated Tregs, protecting pups from dysregulated inflammatory responses upon infection with pneumonia virus of mice (PVM) and, later, from asthma [150]. Although microbiota signals have been attributed mainly to bacterial commensals, the virome was also proposed to determine the functions of visceral pDCs [238].

Visceral pDCs can contribute to inflammatory or metabolic diseases, mainly via IFN production (Fig. 4). In a mouse model of Sjörgen syndrome, numerous IFN-producing pDCs were found in affected salivary glands, and their depletion ameliorated disease [239]. Exposure to a high-fat diet-induced the recruitment of pDCs into visceral adipose tissue (VAT) [240], where IFN-producing pDCs induced the loss of VAT-associated Tregs, promoting obesity [241]. In contrast, in a diabetes model induced by LCMV infection, pancreatic IFN-producing pDCs were protective, as they activated NKT cells that controlled LCMV spreading in the pancreas and hence locally limited CD8+ T-cell activation, leading to reduced tissue damage and insulitis [242]. In patients suffering from colonic inflammatory diseases, pDCs are numerous in the colon, but their contribution to disease is controversial since, in mice, they were reported to be protective [234, 238], deleterious [243] or dispensable [244], depending on the colitis model and on the method used for pDC depletion/targeting. In a *Citrobacter rodentium* colonic infection model, pDCs played a beneficial role, limiting bacterial spreading and ameliorating tissue lesions [245, 246].

### pDCs in skin homeostasis

pDCs are rare in healthy skin [94] but are rapidly recruited to wounded skin, where they promote tissue healing (Fig. 4) [247, 248]. The commensal skin microbiota colonizing damaged skin activates neutrophils to produce CXCL10, which recruits pDCs. Once in the injured skin, pDCs produce IFN-I upon recognition of complexes between CXCL10 and DNA from commensal bacteria, which activates dermal macrophages and skin fibroblasts to promote skin healing. Dysregulation of this pDC-dependent process of epithelial repair likely also contributes to certain inflammatory skin pathologies (Fig. 4), including psoriasis [223, 249] and rosacea [250]. A high frequency of CD123^+^ and/or BDCA2^+^ cells, identified as pDCs, as well as an increased IFN response, were detected in skin biopsies of patients suffering from cutaneous lupus erythematous (CLE) [251], systemic sclerosis [252], vitiligo [253], alopecia areata [254], or several other skin disorders [255]. As pDCs infiltrating inflamed skin are thought to be a major source of IFNs that fuels chronic inflammation and autoimmunity, whether their depletion or functional inhibition with antibodies is beneficial in human skin pathologies is being evaluated in the clinic. Both VIB7734, an anti-ILT7 mAb, and litifilimab, an anti-BDCA2 mAb, significantly reduced pDC frequency and tissue inflammation when used subcutaneously in CLE patients [74, 75, 227]. However, caution must be taken in the interpretation of these results since the BDCA2^+^ cells present in wounded skin include not only pDCs but also cDCs and tDCs [256]. Recent reports have questioned the deleterious contribution of pDCs to CLE, claiming that keratinocytes are the main source of IFNs, whereas the pDCs present in the skin and blood of CLE patients are senescent and unable to produce IFNs [217, 257]. However, it is possible that these senescent pDCs are in a refractory state after their previous in vivo activation of IFN production during early disease onset. Indeed, a fraction of the BDCA2^+^ pDCs present in wounded skin had an activated phenotype [256] compatible with the terminal activation state of IFN-producing pDCs described in mice during systemic MCMV infection [83].

### pDCs in homeostasis of the brain: Role in EAE

In the brain parenchyma, pDCs are absent or very rare at steady state but are more numerous in multiple sclerosis (MS), a chronic inflammatory disease of the central nervous system (CNS) (Fig. 3). MS can be modeled in mice by vaccination against myelin oligodendrocyte glycoprotein (MOG), resulting in the establishment experimental autoimmune encephalomyelitis (EAE), the development of which depends on pathogenic autoreactive Th1 and Th17 cells. pDC depletion upon anti-Bst2 antibody administration can decrease or increase disease severity, depending on whether the agent is administered before or after the onset of EAE (Fig. 4) [258, 259]. After the onset of EAE, pDCs play an immunosuppressive role, mainly by promoting the development of IL-10^+^ Tregs that inhibit pathogenic IL-17^+^ and IFN-γ^+^ autoreactive T cells [259–262]. Indeed, during EAE, the adoptive transfer of MOG-loaded pDCs, which were eventually preactivated with TLR9 ligands, promoted antigen delivery to protective endogenous pDCs recruited into the spinal cord (SC) via a chemerin-dependent pathway [259]. Whether MHC-II expression on pDCs is required for the protective role of SC pDCs during EAE varies depending on the experimental setting [260, 261]. During EAE, specific delivery of IFN-I to SiglecH^+^ cells, including pDCs, by using AcTaferons (activity-on-target IFNs, AFNs) induced immunosuppressive effects and ameliorated EAE even more efficiently when IFN-I was combined with AFN targeted to B cells [263].

### Role of bone marrow pDCs in thrombopoiesis

Platelets are essential regulators of hemostasis and thrombosis. Their generation (thrombopoiesis) occurs in the bone marrow, where they are released into the bloodstream from their immediate precursors, megakaryocytes (MKs). Platelet cell surface desialylation caused by exposure to the Thomsen-Friedenreich antigen (TF antigen) can lead to thrombocytopenia, which can be treated with sialidase inhibitors. Genetic defects in TF sialylation are frequent in pediatric immune thrombocytopenia (ITP) and are often associated with high titers of anti-TF antibodies and increased response to IFNs [264, 265]. Many immune cell types, including pDCs, have been proposed to contribute to thrombocytopenia (Fig. 4) [265]. Mice that were genetically deficient in the ST3 β-galactoside α-2,3-sialyltransferase 1 selectively in MK (St3gal1^MK-/-^) displayed thrombocytopenia and an enrichment of the transcriptomic pDC signature in BM cells [264]. Platelet counts could be partially and transiently rescued in St3gal1^MK-/-^ mice by blocking the response to IFNs or by treatment with an agonistic anti-SiglecH antibody [264] known to inhibit pDC IFN production in response to TLR7/9 triggering [73]. These results suggest that, in the bone marrow, via their C-type lectin receptors, including SiglecH triggering which inhibits IFN production, pDCs monitor changes in the sialylation status of the MK surface. Pathophysiological conditions leading to desialylation of the surface of MKs decrease the triggering of SiglecH and, putatively, other inhibitory C-type lectin receptors in interacting pDCs, relieving the disruption of IFN production, which partially inhibits proplatelet release by MKs. Further studies are required to formally test this hypothesis, including analyses of thrombopoiesis in mice specifically lacking pDCs or in mice with specific inactivation of SiglecH or DAP12 in pDCs, as well as biochemical analyses of the ability of SiglecH to bind the TF antigen. In addition, under which pathophysiological conditions and how the inhibition of thrombopoiesis by pDC IFNs upon desialylation of MKs benefits the host remain to be determined.

## Conclusions and Perspectives

In vivo animal studies, first and foremost in mice but also in a few studies in macaques, pigs or cattle, have shown that splenic or LN pDCs constitute a major source of IFN production during many but not all systemic viral infections. It appears to be also the case for the pDCs present in the draining LNs of the virus inoculation site during peripheral infections, as well as in non-lymphoid barrier tissues, depending on the virus, the tissue, and the host species. However, in most of the models examined, pDCs are seldom a unique source of IFNs, and their depletion, or their inability to produce IFNs, does not strongly compromise the ability of the host to control viral infection, with only a few documented exceptions. This is also the case in humans, in the context of modern hygiene and society, since genetic primary immunodeficiencies compromising IFN production downstream of the endosomal receptors TLR7/9, including pDC IFN production in response to DNA or RNA viruses, do not appear to compromise overall antiviral immunity, with the notable exceptions of respiratory IAV or SARS-CoV-2 infections. Even in the case of respiratory IAV or SARS-CoV-2 infections, it is not clear to what extent the protective effects of IFNs downstream of the TLR7/9-to-MYD88-to-IRF7 signaling pathway depend strictly on pDCs or if IFNs could be produced by other immune cells, including monocytes or macrophages. Mechanistically, under conditions where pDCs are the main source of IFNs in vivo during a viral infection, interfering with their function causes a major decrease in IFN levels in the infected host; however, the low level of IFN production occurring in other cells would be sufficient to promote a general increase in intrinsic antiviral immunity, viral control, and host resistance to infection-induced disease. In other words, pDC IFN production appears to be largely redundant with that of other cell types involved in host antiviral immunity under most of the pathophysiological settings examined thus far. Even more unexpectedly, pDC IFN production could be deleterious for the host in certain chronic or respiratory viral infections, causing a disease largely due to unbridled inflammation and overshooting or miswiring of the immune response, leading to severe damage to vital organs or systems, with detrimental consequences during the viral infection itself or in response to secondary infections by opportunistic pathogens.

Hence, we face a paradox: pDCs are a major source of IFN during many acute primary viral infections, but this function is largely redundant for host resistance and can even be deleterious, contrary to the dogma currently prevailing in the field. This is puzzling considering that the molecular make-up of pDCs and their unique ability to produce high amounts of all IFN subtypes in response to viral-type stimuli are strongly conserved in vertebrates [27, 28]. Therefore, it is likely that pDCs and their IFN production are important from an evolutionary standpoint but in pathophysiological contexts or through mechanisms that still remain to be discovered. Thus, to understand whether, in which pathological context and tissue, and how pDCs exert beneficial, redundant, or deleterious functions, we need to overcome our preconceived ideas and think outside the box. This necessitates considering other functions for pDCs than the rapid reinforcement of antiviral intrinsic immunity at the site of initial infection, considering other possibilities of cellular interactions and on different spatial and temporal scales. This also requires changing experimental readouts to move beyond the measurement of IFN production and of the early control of viral replication during acute infections. One hypothesis could be that pDCs could protect against superinfections by heterologous viruses occurring shortly after the first infection by reinforcing intrinsic immunity in regions of viral entry into the body distant from the site of infection by the first virus. Hence, immune responses to IFNs (e.g., ISG induction) should be measured in various tissues among WT, pDC-deficient, and *Ifnar1*-KO mice, and the immune responses to systemic or local superinfections should be analyzed to examine the possible role of pDCs in immediate cross-protection against other viruses throughout the whole body or specifically in barrier tissues. Another hypothesis is that pDCs promote critical immunoregulatory functions by transporting specific subtypes of IFNs or other signals to the right cell types, in the right place and at the right time. Indeed, whereas virtually any virus-infected cell can produce IFN-β and some IFN-α subtypes, pDCs uniquely produce very high levels of all IFN subtypes without being infected and are highly motile [106]. In this regard, IFN-producing pDCs may play specific and nonredundant roles in shaping innate and adaptive immunity by promoting efficient memory responses to secondary infections. Hence, host resistance and immune memory or trained immunity should be assessed upon late autologous or heterologous challenges compared between WT and pDC-deficient mice. In addition, more studies should be performed to decipher whether, in which tissues, and how pDCs contribute to essential functions at steady state.

Although the importance of pDCs in natural antiviral defense remains an open question, promoting pDC IFN production has been reported to be therapeutically effective in promoting virus control in experimental mouse models of viral infections [266] and in human patients suffering from genital warts [267]. This could also be the case for certain cancers [268, 269]. Moreover, accumulating data strongly support a major role for pDCs in autoimmunity, particularly in lupus erythematosus, not only in mouse models [218, 219] but also in human patients where pDC depletion or the inhibition of IFN production reduces skin lesions [74, 75, 227]. Thus, a better understanding of the cellular and molecular mechanisms controlling pDC IFN production is important for designing novel strategies to manipulate these responses to either enhance or inhibit them depending on the pathophysiological context to promote health and disease. To reach this goal, a number of outstanding questions on pDCs need to be answered (Box 1).

Importantly, advances in the understanding of the physiological role of pDCs have been hampered by the lack of experimental methods enabling specific and penetrant tracking or targeting of these cells in vivo, without off-target effects or artifactual induction of IFNs [206]. This difficulty is explained in part by the fact that mouse pDCs do not express any specific single gene or cell surface receptor, such that it is not possible to target them specifically through a classical knock-in or knock-out strategy or via injection of depleting or inhibitory antibodies (Table 1). We have overcome this bottleneck by designing an intersectional genetic strategy based on the specific coexpression of the *Siglech* and *Pacsin1* genes in pDCs to generate pDC-reporter (SCRIPT) mice (Table 2) [24]. This tool will be critical for deciphering the spatiotemporal dynamics of pDC recruitment and activation for IFN production across tissues, as well as their interactions with other cell types in situ, in different pathophysiological models. More generally, the development of novel methods to specifically deplete pDCs or inhibit their IFN production will be key for future studies. This might be achieved by adapting the design of the SCRIPT mice to replace the tdTomato transgene with the gene encoding the active subunit of the diphtheria toxin to generate pDC-less mice that are constitutively and specifically devoid of pDCs. This type of intersectional genetic strategy might also be exploited to generate a mutant mouse strain expressing Cre recombinase specifically in pDCs to inactivate candidate genes specifically in these cells.
